# Supplementary material for: Vitamin D insufficiency in COVID-19 and influenza A, and critical illness survivors: a cross-sectional study
Source: BMJ Open. 2021 Oct 22;11(10):e055435. doi: 10.1136/bmjopen-2021-055435 (PMC8728359; doi:10.1136/bmjopen-2021-055435)
Supplement: Supplementary data [file bmjopen-2021-055435supp001.pdf]

SUPPLEMENTARY DATA

Supplementary Methods

Definition of vitamin D status

A total 25(OH)D concentration of 50nmol/L is the value widely used to define vitamin D sufficiency since experimental studies have shown that this is the concentration at which parathyroid hormone concentrations plateau [1,2]. Furthermore, based on evidence from the Institute of Medicine (IOM) and the Scientific Advisory Committee on Nutrition (SACN) which demonstrate an increased risk of poor muscoskeletal health with 25(OH)D levels between 20-30 nmol/L, the Royal Osteoporosis Society guidelines (which advice on testing and treatment of vitamin D in primary care in for the NHS), suggest that plasma 25(OH)D of 25-50 nmol/L may be inadequate in some people [3].

1. Sai AJ, Walters RW, Fang X, Gallagher JC. Relationship between Vitamin D, Parathyroid Hormone, and Bone Health. J Clin Endocrinol Metab **2011**; 96:E436–E446. Available at: https://doi.org/10.1210/jc.2010-1886.
2. Lips P. Vitamin D Deficiency and Secondary Hyperparathyroidism in the Elderly: Consequences for Bone Loss and Fractures and Therapeutic Implications. Endocr Rev **2001**; 22:477–501. Available at: https://doi.org/10.1210/edrv.22.4.0437.
3. Francis R, Aspray T, Fraser W, et al. Vitamin D and Bone Health : A Practical Clinical Guideline for Patient Management. 2018. Available at: https://strwebprdmedia.blob.core.windows.net/media/ef2ideu2/ros-vitamin-d-and-bone-health-in-adults-february-2020.pdf.

Supplementary Table 1: LC-MS/MS method parameters

**Parameter**	**COVID-19** (n=259) **/ ICU** (n=139) **sample method**	**Influenza A** (n=93) **/ healthy controls** (n=36) **sample method**
**Sample preparation**		
Isotopically labelled internal standards	d_3-25(OH)D2<br>$^{13}C_5$-25(OH)D3	-<br>d_6-25(OH)D3
Extraction method	Automated SLE	PPT + LLE
Derivatization	DMEQ-TAD	-
**LC-MS instrumentation**		
LC-MS system	Shimadzu Nexera UPLC – Sciex QTrap 6500+	Waters ACQUITY TQD UPLC/MS/MS
LC column	Raptor Fluorophenyl column (2.7µm 100 Å, 100 x 2.1 mm)	Phenyl reversed phase LC column
Ionization mode	ESI, positive	Turbulon Spray, positive
Detection mode	MRM	MRM
**Method specifications**		
LLOD	0.5 nmol/L 25(OH)D2<br>4 nmol/L 25(OH)D3	10 nmol/L 25(OH)D2<br>10 nmol/L 25(OH)D3
Inter-assay precision (CV)	<11.5% 25(OH)D2<br><11.5% 25(OH)D3	<11% 25(OH)D2<br><10% 25(OH)D3

n: number of patient samples analysed; d: deuterium labelled; ^{13}C: carbon 13 labelled; SLE: supported liquid extraction performed on the Biotage® Extrahera™; PPT: protein precipitation; LLE: liquid liquid extraction using n-hexane; ESI: electrospray ionization; MRM: multiple reaction monitoring; LLOD: lower limit of detection; CV: coefficient of variation.

Supplementary Table 2: Multivariable analyses of total 25(OH)D and vitamin D status and outcomes in COVID-19

Variable	Odds ratio	p-value
**Vitamin D status**		
Receipt of IMV		
**Sufficient**[a]	0.26 (0.1-0.62)	**0.004**
Male sex	2.41 (1.18-4.91)	**0.015**
Comorbidity count[b]	-	0.805
Day of illness[b]	-	0.462
Age[b]	-	**0.018**
In-hospital mortality		
**Sufficient**[a]	0.27 (0.11-0.68)	**0.005**
Male sex	2.52 (1.13-5.63)	**0.024**
Comorbidity count[b]		**0.016**
Day of illness[b]	-	0.576
Age[b]	-	0.059
**Total 25(OH)D concentration**		
In-hospital mortality		
Male sex	2.49 (1.12-5.57)	**0.026**
Comorbidity count[b]	-	**0.030**
**Total 25(OH)D**[b]	-	0.068
Day of illness[b]	-	0.495
Age[b]	-	**0.046**

[a]total 25(OH)D >50 nmol/l
[b]smoothed

Supplementary Table 3: Multivariable analysis of total 25(OH)D concentration and in-hospital mortality in influenza A

Variable	Odds ratio	p-value
In-hospital mortality		
Male sex	0.84	0.798
Comorbidity count[a]	-	0.539
**Total 25(OH)D**[a]	-	0.421
Day of illness[a]	-	0.244
Age[a]	-	0.200

[a]smoothed

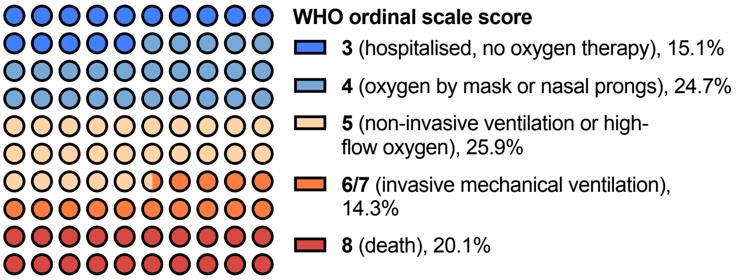

Supplementary Figure 1: WHO COVID-19 ordinal severity scale scores
The % refers to the % of patients in the cohort (n=259) with the score. Scores represent maximum illness severity during the hospital admission.

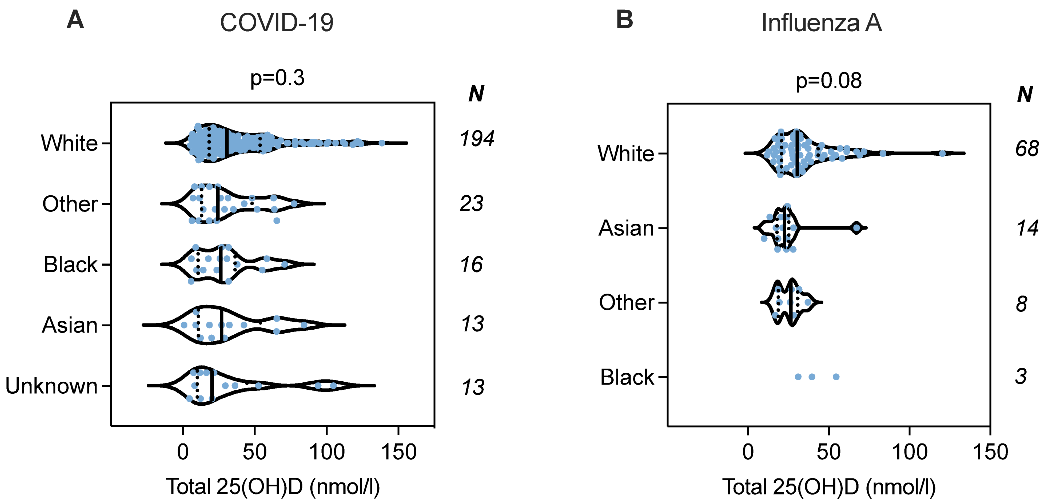

Supplementary Figure 2: Total 25(OH)D concentration stratified by ethnicity
(A) COVID-19 and **(B)** influenza A. The solid line within the violin plot represents the median and the dotted lines represent the interquartile range. Groups ≤5 are shown as individual data points. Groups were compared by ANOVA. N refers to the number of patients in each group.

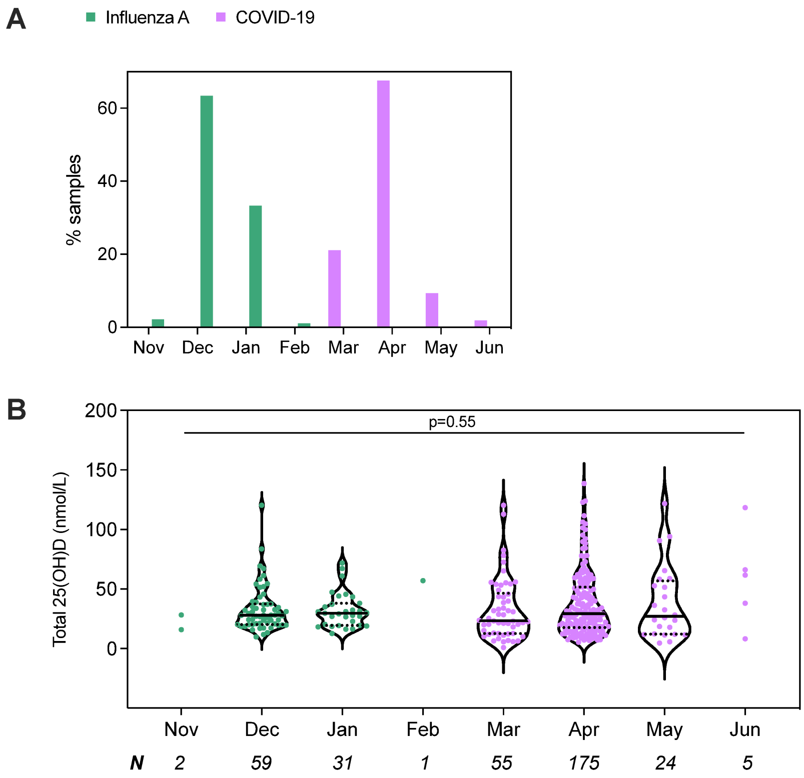

Supplementary Figure 3: Total 25(OH)D stratified by months of the year
(A) Month of the year during which samples were obtained from people with influenza A (2009-2011) and COVID-19 (2020). **(B)** Total 25(OH)D concentrations stratified by month of the year the sample was obtained. Groups compared by Kruskal-Wallis test. The solid line within the violin plot represents the median and the dotted lines represent the interquartile range. N refers to the number of samples for each month. Groups ≤5 are shown as individual data points.

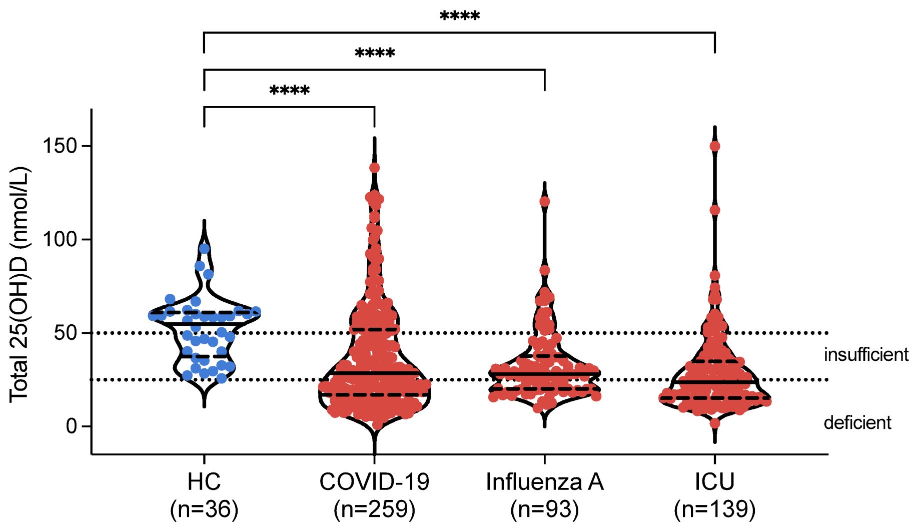

Supplementary Figure 4: Total 25(OH)D in patient cohorts compared to healthy controls
Total 25(OH)D measured in healthy controls ("HC", from the MOSAIC study recruited between June-September 2011), hospitalised patients with COVID-19 and influenza A, and non-selected critical illness survivors ("ICU"). The solid line within the violin plot represents the median and the dashed lines represent the interquartile range. Patient cohorts compared to healthy controls by Kruskal-Wallis test and Dunn's multiple comparisons test. **** p<0.0001

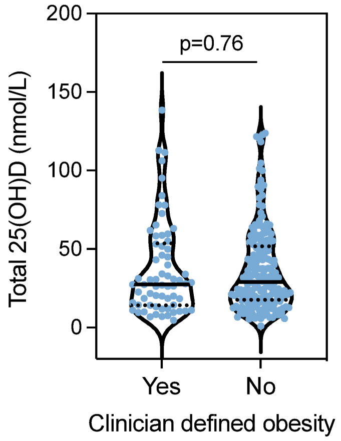

Supplementary Figure 5: Total 25(OH)D in patients with COVID-19 with/without obesity
Total 25(OH)D levels in hospitalised patients with COVID-19 with/without clinician defined obesity. Groups compared by Mann-Whitney test. The solid line within the violin plot represents the median and the dotted lines represent the interquartile range.

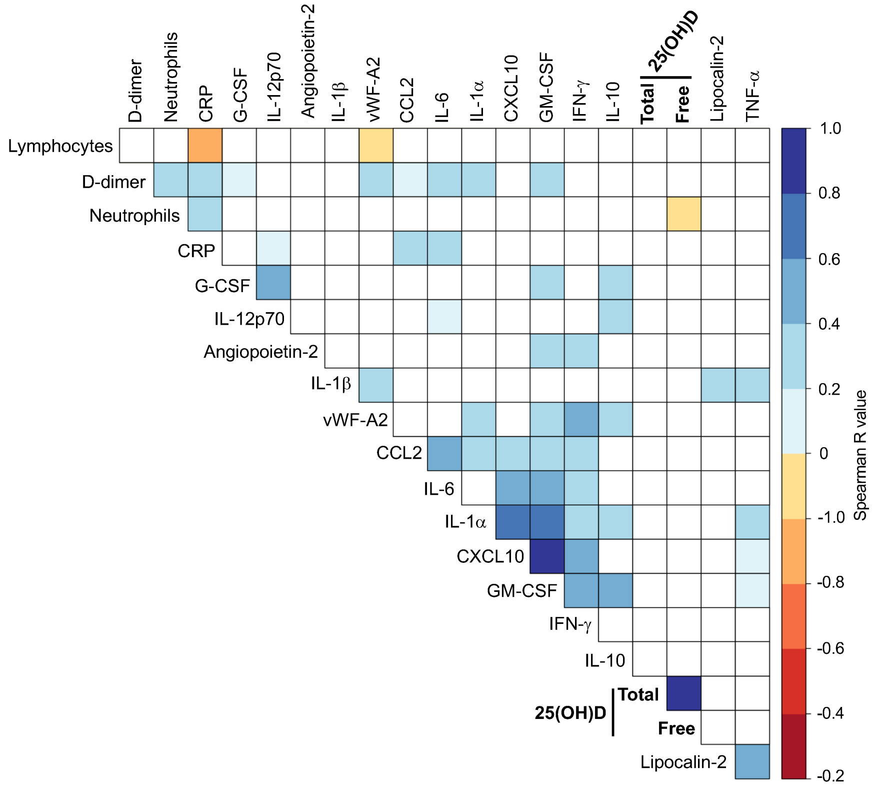

Supplementary Figure 6: Correlation analysis of 25(OH)D and inflammatory mediators

Correlogram of concentrations of plasma inflammatory mediators associated with COVID-19 severity and 25(OH)D (free and total). Cells with a correlation with p<0.05 (after correction for multiple comparisons) are shaded according to the Spearman R value. Inflammatory mediator measurements were available for 66 patients. Analysis was performed using the *corrplot* package in R.

CRP: C-reactive protein; G-CSF: granulocyte colony-stimulating factor; IL: interleukin; vWF: von Willebrand Factor; CCL2: C-C Motif Chemokine Ligand 2; CXCL10: C-X-C Motif Chemokine Ligand 10; GM-CSF: granulocyte-macrophage colony-stimulating factor; IFN-γ: interferon gamma; TNF-α: tumour necrosis factor alpha.

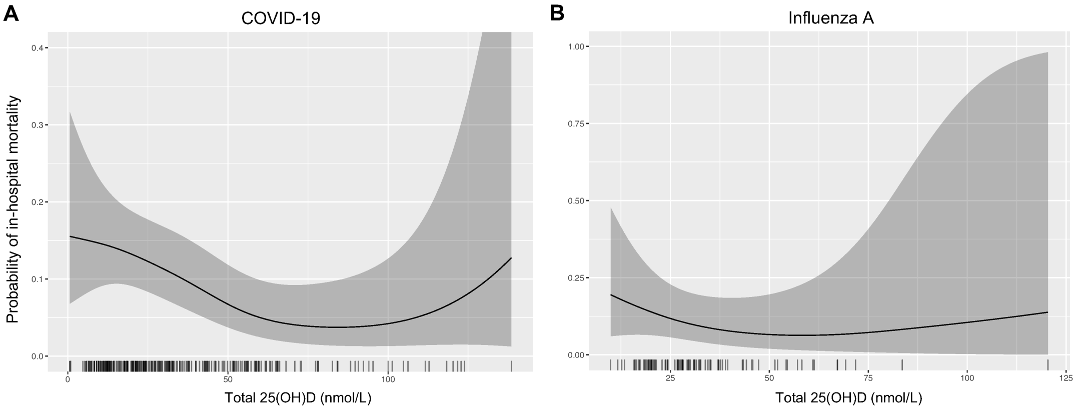

Supplementary Figure 7: Total 25(OH)D concentration and in-hospital mortality in COVID-19 and influenza A.

Smoothed predicted probability of in-hospital mortality vs. total 25(OH)D concentration (with other co-variates at mean values) from the binary logistic regression multivariable models for hospitalised people with **(A)** COVID-19 and **(B)** influenza A. Grey ribbon represents estimated 95% confidence interval and the x-axis ticks show observations.
