# Supplementary material for: Vitamin D insufficiency in COVID-19 and influenza A, and critical illness survivors: a cross-sectional study
Source: BMJ Open. 2021 Oct 22;11(10):e055435. doi: 10.1136/bmjopen-2021-055435 (PMC8728359; doi:10.1136/bmjopen-2021-055435)
Supplement: Author's manuscript [file bmjopen-2021-055435.draft_revisions.pdf]

BMJ Open

BMJ Open is committed to open peer review. As part of this commitment we make the peer review history of every article we publish publicly available.

When an article is published we post the peer reviewers' comments and the authors' responses online. We also post the versions of the paper that were used during peer review. These are the versions that the peer review comments apply to.

The versions of the paper that follow are the versions that were submitted during the peer review process. They are not the versions of record or the final published versions. They should not be cited or distributed as the published version of this manuscript.

BMJ Open is an open access journal and the full, final, typeset and author-corrected version of record of the manuscript is available on our site with no access controls, subscription charges or pay-per-view fees (http://bmjopen.bmj.com).

If you have any questions on BMJ Open's open peer review process please email info.bmjopen@bmj.com

BMJ Open

Vitamin D insufficiency in COVID-19, influenza A and critical illness survivors: a cross-sectional study

Journal:	*BMJ Open*
Manuscript ID	bmjopen-2021-055435
Article Type:	Original research
Date Submitted by the Author:	13-Jul-2021
Complete List of Authors:	Hurst, Emma A.; University of Edinburgh<br>Mellanby, Richard; The University of Edinburgh<br>Handel, Ian; University of Edinburgh<br>Griffith, David; Royal Infirmary of Edinburgh, Anaesthesia, Critical Care and Pain<br>Rossi, Adriano; University of Edinburgh MRC Centre for Inflammation Research, MRC Centre for Inflammation Research<br>Walsh, Timothy; The University of Edinburgh<br>Shankar-Hari, Manu; Guys and Guy's and St Thomas' Hospital NHS Foundation Trust, Intensive Care Unit; Kings College, London, Division of Asthma, Allergy and Lung Biology<br>Dunning, Jake; University of Oxford<br>Homer, Natalie Z.; University of Edinburgh<br>Denham, Scott G.; University of Edinburgh<br>Devine, Kerri; University of Edinburgh<br>Holloway, Paul A.; Imperial College London, Department of Metabolism, Digestion and Reproduction<br>Moore, Shona C.; University of Liverpool<br>Thwaites, Ryan S.; Imperial College London<br>Samanta, Romit J.; University of Cambridge, Department of Medicine<br>Summers, Charlotte; University of Cambridge, Department of Medicine<br>Hardwick, Hayley E.; University of Liverpool<br>Oosthuyzen, Wilna; University of Edinburgh<br>Turtle, Lance; University of Liverpool, NIHR Health Protection Research Unit in emerging and zoonotic infections, Institute of Infection, Veterinary and Ecological Sciences; Liverpool University Hospitals NHS Foundation Trust, Tropical and Infectious Disease Unit<br>Semple, Malcolm G; University of Liverpool, Institute of Translational Medicine<br>Openshaw, PJ; Imperial College London<br>Baille, J; Roslin Institute, Division of Genetics and Genomics<br>Russell, Clark; University of Edinburgh MRC Centre for Inflammation Research
Keywords:	COVID-19, INTENSIVE & CRITICAL CARE, Respiratory infections < THORACIC MEDICINE

I, the Submitting Author has the right to grant and does grant on behalf of all authors of the Work (as defined in the below author licence), an exclusive licence and/or a non-exclusive licence for contributions from authors who are: i) UK Crown employees; ii) where BMJ has agreed a CC-BY licence shall apply, and/or iii) in accordance with the terms applicable for US Federal Government officers or employees acting as part of their official duties; on a worldwide, perpetual, irrevocable, royalty-free basis to BMJ Publishing Group Ltd ("BMJ") its licensees and where the relevant Journal is co-owned by BMJ to the co-owners of the Journal, to publish the Work in this journal and any other BMJ products and to exploit all rights, as set out in our licence.

The Submitting Author accepts and understands that any supply made under these terms is made by BMJ to the Submitting Author unless you are acting as an employee on behalf of your employer or a postgraduate student of an affiliated institution which is paying any applicable article publishing charge ("APC") for Open Access articles. Where the Submitting Author wishes to make the Work available on an Open Access basis (and intends to pay the relevant APC), the terms of reuse of such Open Access shall be governed by a Creative Commons licence – details of these licences and which Creative Commons licence will apply to this Work are set out in our licence referred to above.

Other than as permitted in any relevant BMJ Author's Self Archiving Policies, I confirm this Work has not been accepted for publication elsewhere, is not being considered for publication elsewhere and does not duplicate material already published. I confirm all authors consent to publication of this Work and authorise the granting of this licence.

For peer review only

Vitamin D insufficiency in COVID-19, influenza A and critical illness survivors: a cross-sectional study

Emma A. Hurst[1,2], Richard J. Mellanby[1], Ian Handel[1], David M. Griffith[3], Adriano G. Rossi[4], Timothy S. Walsh[4], Manu Shankar-Hari[5], Jake Dunning[6], Natalie Z. Homer[2], Scott G. Denham[2], Kerri Devine[2], Paul A. Holloway[7], Shona C. Moore[8], Ryan S. Thwaites[9], Romit J. Samanta[10], Charlotte Summers[10], Hayley E. Hardwick[8], Wilna Oosthuyzen[11], Lance Turtle[7,12], Malcolm G. Semple[8,13], Peter J.M. Openshaw[9], J. Kenneth Baillie[11] and Clark D. Russell[4,11], on behalf of the ISARIC4C investigators

[1]The Roslin Institute and Royal (Dick) School of Veterinary Studies, University of Edinburgh, Edinburgh, U.K.

[2]Mass Spectrometry Core, Edinburgh Clinical Research Facility, Queen's Medical Research Institute, University of Edinburgh, Edinburgh, U.K.

[3]Molecular, Genetic and Population Health Sciences, University of Edinburgh, Edinburgh, U.K.

[4]University of Edinburgh Centre for Inflammation Research, The Queen's Medical Research Institute, Edinburgh, U.K.

[5]Peter Gorer Department of Immunobiology, School of Immunology & Microbial Sciences, Kings College London, U.K.

[6]Centre for Tropical Medicine and Global Health, University of Oxford, Oxford, U.K.

[7]Faculty of Medicine, Department of Metabolism, Digestion and Reproduction, Imperial College London, U.K.

[8]NIHR Health Protection Research Unit in Emerging and Zoonotic Infections, Institute of Infection, Veterinary and Ecological Sciences, Faculty of Health and Life Sciences, University of Liverpool, Liverpool, U.K.

[9]National Heart and Lung Institute, Imperial College London, U.K.

[10]Department of Medicine, University of Cambridge, U.K.

[11] Division of Genetics and Genomics, Roslin Institute, University of Edinburgh, Edinburgh, U.K.

For peer review only - http://bmjopen.bmj.com/site/about/guidelines.xhtml

[12]Tropical and infectious disease unit, Liverpool University Hospitals NHS Foundation Trust (member of Liverpool Health Partners), U.K.

[13]Respiratory Medicine, Alder Hey Children's Hospital, Liverpool, U.K.

Corresponding author:

Clark D Russell. University of Edinburgh Centre for Inflammation Research, The Queen's Medical Research Institute, 47 Little France Crescent, Edinburgh, U.K. EH16 4TJ. clark.russell@ed.ac.uk

Running title: Vitamin D in COVID-19, IAV & critical illness

Word counts:

Abstract: 266

Main: 2998

For peer review only

ABSTRACT

Objectives: The steroid hormone vitamin D has roles in immunomodulation and bone health. Insufficiency is associated with susceptibility to respiratory infections. We report 25(OH)D measurements in hospitalised people with COVID-19 and influenza A, and survivors of critical illness, to test the hypotheses that vitamin D insufficiency scales with illness severity and persists in survivors.

Design: Cross-sectional study

Setting and Participants: Plasma was obtained from 295 hospitalised people with COVID-19 (ISARIC/WHO CCP-UK study), 93 with influenza A (MOSAIC study, during the 2009-10 H1N1 pandemic), and 139 survivors of non-selected critical illness (prior to COVID-19 pandemic). Total 25(OH)D was measured by liquid chromatography-tandem mass spectrometry. Free 25(OH)D was measured by ELISA in COVID-19 samples.

Outcome measures: Receipt of invasive mechanical ventilation (IMV) and in-hospital mortality.

Results: Vitamin D insufficiency (total 25(OH)D 25-50 nmol/L) and deficiency (<25nmol/L) were prevalent in COVID-19 (29.3% and 44.4% respectively), influenza A (47.3% and 37.6%) and critical illness survivors (30.2% and 56.8%). In COVID-19 and influenza A, total 25(OH)D measured early in illness was lower in patients who received IMV (19.6 vs. 31.9 nmol/L, $p<0.0001$ and 22.9 vs. 31.1 nmol/L, $p=0.0009$ respectively). In COVID-19, biologically-active free 25(OH)D correlated with total 25(OH)D, was lower in patients who received IMV, but was not associated with selected circulating inflammatory mediators.

Conclusions: Vitamin D deficiency/insufficiency was present in the majority of hospitalised patients with COVID-19 or influenza A, correlated with severity and persisted in critical illness survivors at concentrations expected to disrupt bone metabolism. These findings support early supplementation trials to determine if insufficiency is causal in progression to severe disease, and investigation of longer-term bone health outcomes.

KEYWORDS: Vitamin D; Free 25-hydroxyvitamin-D; COVID-19; critical illness; influenza A.

For peer review only

STRENGTHS AND LIMITATIONS OF THIS STUDY

- We report 25(OH)D liquid chromatography-tandem mass spectrometry measurements in well characterised hospitalised people with COVID-19, influenza A and survivors of non-selected critical illness.
- For the first time, we report measurement of biologically active free 25(OH)D in addition to total in COVID-19.
- Samples from people with COVID-19 and influenza A were obtained early in the course of disease.
- The association of 25(OH)D with outcomes in COVID-19 and influenza A was assessed with binary logistic regression multivariable models to correct for other known relevant covariates.
- The observational nature of the study means it is not clear whether vitamin D status led to poor clinical outcome or was a consequence of illness severity.

For peer review only

INTRODUCTION

Vitamin D metabolites contribute to bone metabolism, calcium homeostasis and immunomodulation. Vitamin D is a steroid pre-pro-hormone which is converted to the main circulating form 25-hydroxy-vitamin D (25(OH)D), and subsequently to the active hormone 1,25 dihydroxy-vitamin D (1,25(OH)$_2$D). This second activation step occurs in the kidney, modulated by parathyroid hormone (PTH), for "endocrine" calciotropic effects, and also under local control within extra-renal tissues, including immune cells, for direct action. These "intracrine" actions on immune cells mediate anti-microbial and anti-inflammatory effects [1]. The majority of 25(OH)D circulates bound to proteins, principally vitamin D binding protein (85-90%), and the relatively small unbound ("free") fraction is available to immune cells [2].

In the context of infectious diseases, vitamin D insufficiency (routinely determined by total 25(OH)D measurement) is associated with increased incidence and severity of respiratory tract infections [3–5] including coronavirus disease 2019 (COVID-19) [6–7]. A geographic association between vitamin D deficiency prevalence and COVID-19 incidence and mortality has been reported [8]. Free 25(OH)D has not yet been investigated in COVID-19, but this is required to fully understand vitamin D homeostasis and any effects on systemic inflammation. Clinical trials of vitamin D supplementation in respiratory diseases have returned mixed results [9–12]. Potential beneficial effects of vitamin D supplementation may be pathogen-specific, and dependent upon timing and route of administration. In addition to an interest in modifying acute illness outcomes, longer term effects on bone health warrant consideration as critical illness is associated with loss of bone mineral density after recovery [13].

In this cross-sectional study, we report measurements of total and free 25(OH)D in hospitalised people with COVID-19, and total 25(OH)D in hospitalised people with influenza A and survivors of critical

For peer review only - http://bmjopen.bmj.com/site/about/guidelines.xhtml

illness. We use these three datasets to test the hypotheses that vitamin D insufficiency in severe respiratory virus infections scales with severity and persists in survivors of critical illness.

For peer review only

For peer review only - http://bmjopen.bmj.com/site/about/guidelines.xhtml

METHODS

Patients and sampling

COVID-19

The ISARIC WHO Clinical Characterization Protocol for Severe Emerging Infections in the UK (CCP-UK) is an ongoing prospective cohort study of hospitalized patients with COVID-19, which is recruiting in 308 hospitals in England, Scotland, Wales and Northern Ireland (National Institute for Health Research Clinical Research Network Central Portfolio Management System ID: 14152), delivered by the ISARIC Coronavirus Clinical Characterisation Consortium (ISARIC4C) investigators. The protocol, revision history, case report form and consent forms are available online at isaric4c/net. The ISARIC/WHO CCP-UK study was registered at https://www.isrctn.com/ISRCTN66726260 and designated an Urgent Public Health Research Study by the National Institute for Health Research UK. A prespecified case report form was used to collect data on patient characteristics, medical interventions received and outcomes, as previously reported [14].

Influenza A

Hospitalised patients with influenza A were recruited between 2009 and 2010 (the first and second H1N1 pandemic waves) and 2011 (the first post-pandemic season) by the MOSAIC (Mechanisms of Severe Acute Influenza Consortium) investigators.

Non-selected critical illness survivors

For peer review only

We include a post-hoc analysis of the RECOVER trial of intensive rehabilitation after critical illness [15]. Full eligibility criteria have been published previously; briefly, adults were recruited who had received invasive mechanical ventilation (IMV) for at least 48 hours and were considered well enough for discharge from the ICU. Patients gave additional consent for participation in a biomarker sub-study and blood samples were collected at ICU discharge [16].

Ethics Approvals

Ethical approval for the ISARIC/WHO CCP-UK study (COVID-19) was given by the South Central Oxford C Research Ethics Committee in England (13/SC/0149), the Scotland A Research Ethics Committee (20/SS/0028), and the WHO Ethics Review Committee (RPC571 and RPC572, 25 April 2013). Ethical approval for the MOSAIC study (influenza A) was given by the NHS National Research Ethics Service, Outer West London Research Ethics Committee (09/H0709/52, 09/MRE00/67), as previously reported [17]. Participants gave informed consent.

LC-MS/MS methods for total 25(OH)D analysis

EDTA plasma concentrations of 25(OH)D2 and 25(OH)D3 isoforms were measured by liquid chromatography tandem mass spectrometry (LC-MS/MS) and summed to derive the total 25(OH)D concentrations presented in the results. For patients with COVID-19 and critical illness survivors, analysis was performed by the Vitamin D Animal Laboratory (VitDAL) using an assay which has been certified as proficient by the international Vitamin D Quality Assessment Scheme (DEQAS) and described in detail in an earlier manuscript, using 200μL plasma [18]. For patients with influenza A, analysis was performed using another LC-MS/MS method at a separate clinical biochemistry laboratory. Full LC-MS/MS methods are presented in Supplementary Table 1.

For peer review only - http://bmjopen.bmj.com/site/about/guidelines.xhtml

Definition of vitamin D status

In addition to the absolute total 25(OH)D concentration, the relationship between vitamin D status and outcomes is often explored using a total 25(OH)D cut off of 50 nmol/L to define populations that are vitamin D sufficient [19]. In this study, total 25(OH)D >50nmol/L is reported as “sufficient”, 25–50 nmol/L as “insufficient” and <25 nmol/L as “deficient” (see Supplementary Methods).

For peer review only

Free 25(OH)D ELISA

Free 25(OH)D was measured using the Free 25OH Vitamin D ELISA (DIAsource ImmunoAssays® S.A, Belgium), following manufacturer’s instructions, using 10µL of serum. Absorbance was measured at 450nm against a reference filter set at 630nm using the Tecan Sunrise™ Microplate Reader (TECAN). GraphPad Prism (version 7.0e for Mac OS X) was used to perform a 4-parameter logistic function to create the calibration curve in order to read the mean concentration of duplicate samples.

Statistical analysis

For univariable analyses, the Shapiro-Wilk test was used to test for normal data distribution then appropriate tests, specified in the text, were used for comparisons. Associations between covariates and outcomes in COVID-19 and influenza A were assessed with binary logistic regression multivariable models. Sex, age, illness duration at time of sampling and comorbidity count were chosen as covariates. The comorbidity count was derived from the same comorbidities (Table 1) from the two cohorts. To allow for potential non-linear relationship between predictors and the probability of an outcome, the models included smoothed thin plate regression spline terms for age, illness duration at time of sampling, comorbidity count and 25(OH)D concentrations. Multivariable models were estimated using the *gam()* function of the R *mgcv* package using the default, thin plate regression

For peer review only - http://bmjopen.bmj.com/site/about/guidelines.xhtml

smoothers [20,21]. The upper limit of smoother dimensionality was set to 9 for all variables excluding the comorbidity count where it was set to 7 as this variable was discrete with 7 levels. Smoother parameters were estimated with restricted maximum likelihood. 25(OH)D concentrations were below the limit of detection (LOD) for 92 patients (free) and 2 patients (total) in the COVID-19 cohort. For the regression models, 25(OH)D values for these patients were imputed as the LOD for the relevant analyte divided by the square root of two [22]. As this is a commonly used but arbitrary method the regression analysis was repeated using zero and the limit of detection as imputed values to assess sensitivity of the result to this assumption. Effects for categorical covariates are reported as odds ratios; smoothed continuous covariates are reported graphically. Statistical analyses were conducted in R [23] using the *mgcv*, *tidyverse* and *gratia* packages.

For peer review only

RESULTS

Patient characteristics

Samples were obtained from 259 people hospitalised due to COVID-19 and 93 people hospitalised due to influenza A. Samples were also obtained from 139 critical illness survivors (prior to the COVID-19 pandemic) at time of ICU discharge. Patient characteristics, including sampling time after symptom onset, are shown in Table 1. For COVID-19 patients, samples were obtained a median of 3 days (IQR 2-6) after hospital admission. Patients with influenza A were younger, more likely to be female and more likely to have asthma compared to the other cohorts. Receipt of IMV and in-hospital mortality did not differ between COVID-19 and influenza A. Details on ethnicity were available for the COVID-19 and influenza A cohorts. No differences in total 25(OH)D were observed between ethnic groups, but only small numbers of participants were from non-white groups (COVID-19 65/259, influenza A 25/93; Supplementary Figure 1). All samples from people with influenza A were collected between the months of November to February (63.4% in December), whereas all samples from people with COVID-19 were collected between March to June (67.6% in April). However, the distribution of total 25(OH)D measurements did not differ when stratified by month (Supplementary Figure 2). Total 25(OH)D concentration was lower in all three patient cohorts when compared to healthy controls (n=36; Supplementary Figure 3).

For peer review only

Table 1: Characteristics of included patients

Characteristic	COVID-19 (n=259)	Influenza A (n=93)	Critical illness survivors (n=139)	p-value[a]
**Demographics**				
Age at admission, years[b]	63 (52-73)	43 (29-50)	63 (53-70)	**<0.0001**
Male sex	175 (67.6)	47 (50.5)	85 (61.2)	0.01
Day of illness at time of sampling[b]	10 (6-16)	7 (4-11)	11 (6-18)[c]	**<0.001**[d]
**Co-morbidities**				
Diabetes mellitus	66 (25.5)	10 (10.8)	23 (16.5)	**0.005**
Chronic cardiac disease	57 (22.4)	17 (18.3)	15 (10.8)	**0.02**
Obesity, clinician defined	44 (18.7)	23 (24.7)	28 (20.1)	0.3
Asthma	41 (16.1)	33 (35.5)	26 (18.7)	**0.0002**
Chronic lung disease, not asthma	35 (13.8)	12 (12.9)	24 (17.3)	0.5
Chronic kidney disease	25 (9.9)	4 (4.3)	NA	0.1
Neoplasia	14 (5.6)	9 (9.7)	NA	0.2
Moderate or severe liver disease	3 (1.2)	4 (4.3)	NA	0.08
**Illness severity**				
Admission to critical care	106 (40.9)	32 (34.4)	139 (100)	0.3[d]
Invasive mechanical ventilation	67 (25.9)	29 (31.2)	139 (100)	0.3[d]
In-hospital mortality	52 (20.1)	12 (12.9)	4 (2.9)[e]	0.2[d]
**Total 25(OH)D status**				
Sufficient (>50 nmol/L)	68 (26.3)	14 (15.1)	18 (12.9)	
Insufficient (25-50 nmol/L)	76 (29.3)	44 (47.3)	42 (30.2)	0.0002
Deficient (<25 nmol/L)	115 (44.4)	35 (37.6)	79 (56.8)	

Data are number (%) unless otherwise stated.

[a]Kruskal-Wallis, Mann-Whitney or Chi^2 test as appropriate

[b]median (interquartile range)

[c]length of ICU stay

[d]comparing COVID-19 and influenza A

[e]death after discharge from ICU

For peer review only - http://bmjopen.bmj.com/site/about/guidelines.xhtml

Total 25(OH)D correlates with severity in COVID-19

The majority of COVID-19 patients had total 25(OH)D concentrations indicative of vitamin D insufficiency (29.3%) or deficiency (44.4%; Table 1). Total 25(OH)D was lower in men than women (median 26.8 [IQR 14.1-47.4] vs. 31.7 [20.1-63.8] nmol/L, p=0.01) and weakly positively correlated with increased age (Pearson *r* 0.25, p<0.0001).

When stratified by receipt of IMV as a marker of illness severity, total 25(OH)D differed significantly with a median concentration of 19.6 nmol/L (IQR 12.6-32.3) in patients receiving IMV compared to 31.9 nmol/L (IQR 20.0-58.3) in the remainder of the cohort (p<0.0001, Figure 1A). When total 25(OH)D was stratified by associated vitamin D status, patients receiving IMV were more likely to be insufficient/deficient (Figure 1A). Amongst patients who received IMV, 64.2% (43/67) were deficient and 26.9% (18/67) were insufficient. Total 25(OH)D concentration was also associated with in-hospital mortality (median 23.2 nmol/L [IQR 15.4-39.9] in non-survivors vs. 29.5 nmol/L [IQR 17.2-55.4] in survivors, p=0.01).

Obesity is a risk factor for severity and mortality in COVID-19, and can be associated with vitamin D deficiency [14]. However, there was no difference in total 25(OH)D concentration between patients with/without clinician defined obesity (Supplementary Figure 4). Inflammatory mediator measurements had previously been performed on plasma samples from 66 patients included in this study [24]. Correlation matrix analysis demonstrated that total 25(OH)D was not significantly associated with circulating markers of systemic inflammation demonstrated to be involved in COVID-19 pathogenesis (Supplementary Figure 5).

Multivariable analyses confirmed that total 25(OH)D concentration and vitamin D status were both independently and negatively associated with receipt of IMV (Table 2, Figure 2A, Supplementary Table

For peer review only - http://bmjopen.bmj.com/site/about/guidelines.xhtml

2). Two patients had total 25(OH)D concentrations below the LOD; using zero and LOD, instead of LOD divided by the square root of two, had no substantive effect on significance of covariates or their effect sizes. Vitamin D status was also independently associated with in-hospital mortality, but total 25(OH)D concentration was not (Supplementary Table 2; Supplementary Figure 6A).

For peer review only

Table 2: Multivariable analyses of 25(OH)D concentration and outcomes

Variable	Odds ratio	p-value
**Total 25(OH)D**		
COVID-19: receipt of IMV		
Male sex	2.33 (1.13-4.78)	**0.022**
Comorbidity count[a]	-	0.487
**Total 25(OH)D**[a]	-	**0.001**
Day of illness[a]	-	0.386
Age[a]	-	0.061
Influenza A: receipt of IMV		
Male sex	2.22 (0.54 – 9.06)	0.27
Comorbidity count[a]	-	0.15
**Total 25(OH)D**[a]	-	**0.016**
Day of illness[a]	-	**0.001**
Age[a]	-	0.19
**Free 25(OH)D**		
COVID-19: receipt of IMV		
Male sex	2.53 (1.24-5.314)	**0.011**
Comorbidity count[a]	-	0.605
**Free 25(OH)D**[a]	-	**0.006**
Day of illness[a]	-	0.577
Age[a]	-	0.053
COVID-19: in-hospital mortality		
Male sex	2.78 (1.25-6.17)	**0.012**
Comorbidity count[a]	-	**0.022**
**Free 25(OH)D**[a]	-	**0.025**
Day of illness[a]	-	0.795
Age[a]	-	**0.041**

IMV: invasive mechanical ventilation

[a]smoothed

For peer review only

Total 25(OH)D correlates with severity in influenza A

We then extended these observations to total 25(OH)D concentrations measured in people hospitalised with influenza A. Total 25(OH)D was not associated with age (p=0.1) or sex (p=0.8). Similar to our findings in COVID-19, the majority of patients had total 25(OH)D concentrations indicative of vitamin D insufficiency (47.3%) or deficiency (37.6%; Table 1). When stratified by receipt of IMV, total 25(OH)D was lower in patients receiving IMV (median 22.9 nmol/L, IQR 18.0-29.8) compared to the remainder of the cohort (median 31.1 nmol/L, IQR 23.8-45.2, p=0.0009) and these patients were more likely to be vitamin D insufficient/deficient (Figure 1B). Total 25(OH)D was lower in non-survivors compared to survivors (median 22.1 nmol/L [IQR17.6-34.1] vs. 29.2 nmol/L [IQR 20.6-38.5]) but this was not statistically significant (p=0.2). Multivariable analysis confirmed an independent negative association between total 25(OH)D and receipt of IMV but not in-hospital mortality (Figure 2B, Table 2; Supplementary Table 3; Supplementary Figure 6B).

Vitamin D deficiency persists in survivors of critical illness

In survivors of non-selected critical illness, at the time of ICU discharge the median total 25(OH)D concentration was 22.9 nmol/L (IQR 14.6-34.6), similar to concentrations in patients with COVID-19/influenza A who required IMV or did not survive. The majority of patients had total 25(OH)D concentrations indicative of vitamin D deficiency (56.8%) or insufficiency (30.2%; Figure 1C and Table 1). Total 25(OH)D concentration was not associated with age (p=0.7), sex (p=0.7) or length of ICU stay (p=0.8). Measurements were not available from earlier in these patients' illnesses.

Free 25(OH)D correlates with severity in COVID-19

For peer review only - http://bmjopen.bmj.com/site/about/guidelines.xhtml

In patients with COVID-19, we found a strong correlation between free and total 25(OH)D concentrations (*r*=0.79, p<0.0001) (Figure 3A). Free 25(OH)D was lower in patients receiving IMV (median 2.4 pg/mL [IQR 2.4-3.4] vs. 3.6 pg/mL [IQR 2.4-5.7], p<0.0001; Figure 3B) but was not statistically different between survivors and non-survivors on univariable analysis (median 2.8 pg/mL [IQR 2.4-4.4] vs. 3.3 pg/mL [IQR 2.4-5.3], p=0.2). In multivariable analysis, free 25(OH)D was negatively associated with both receipt of IMV and in-hospital mortality (Figure 2B-D, Table 2). Free 25(OH)D was not associated with plasma inflammatory mediator concentrations (Supplementary Figure 5).

For peer review only

DISCUSSION

Vitamin D insufficiency was prevalent and scaled with severity in patients with COVID-19 and influenza A, and insufficiency persisted in survivors of critical illness. 73% of COVID-19 patients, 84% of influenza A patients and 87% of critical illness survivors were vitamin D insufficient/deficient, determined by total 25(OH)D measurement. We demonstrate evidence of a strong association between vitamin D status (insufficiency/deficiency) and both COVID-19 severity (receipt of IMV) and in-hospital mortality, with relevant confounders such as sex, age, comorbidities and day of illness adjusted for. This observation was replicated in influenza A but the smaller sample size (n=93 compared to 259) limited multivariable analyses. For the first time, we demonstrate a similar strong negative association between free 25(OH)D and COVID-19 disease severity and mortality. The results from this study extend earlier findings from other observational studies reporting associations between vitamin D status and SARS-CoV-2 infection and COVID-19 outcome [6,7,25–27].

Vitamin D may beneficially modulate the host response against SARS-CoV-2 via intracrine immune signalling. Vitamin D enhances intracellular pathogen clearance, primarily via the induction of autophagy [28]. Importantly, the ability of macrophages to produce cathelicidin, which has anti-viral activity against influenza virus and respiratory syncytial virus, correlates with circulating 25(OH)D concentrations [29]. Although anti-viral effects of vitamin D have not yet been demonstrated *in vitro* for SARS-CoV-2, they have been demonstrated for other bacterial and viral pathogens [30,31]. Consistent with vitamin D having a role in local immunomodulation, neither free nor total 25(OH)D correlated with circulating markers of systemic inflammation involved in COVID-19 pathogenesis (including CRP, IL-6 and GM-CSF).

Evidence for the importance of free versus total 25(OH)D in relation to the mechanisms by which vitamin D exerts antimicrobial and anti-inflammatory functions have been demonstrated [32,33]. We

For peer review only - http://bmjopen.bmj.com/site/about/guidelines.xhtml

now demonstrate that free 25(OH)D was negatively associated with COVID-19 severity and in-hospital mortality. Studies directly measuring free 25(OH)D and immune responses to infection or during critical illness are limited. In a study of 30 critically ill patients, Han et al (2017) showed that supplementation with high dose vitamin D increased free 25(OH)D and plasma cathelicidin concentrations [34]. Another study of 30 patients with sepsis reported similar results when they examined the effects of vitamin D supplementation on bioavailable (combined albumin-bound and free fraction) 25(OH)D and cathelicidin concentrations [35]. Together, these findings suggest that low concentrations of free 25(OH)D may reduce the vitamin D-induced antimicrobial and anti-inflammatory response, compromising immune defences.

We found that 30.2% of patients surviving critical illness and requiring IMV (prior to the COVID-19 pandemic) were vitamin D insufficient and 56.8% were deficient. Vitamin D deficiency is common in critical illness with a reported prevalence of between 40-70% in observational studies of both adults and children worldwide [36,37]. Although some patients may enter ICU in a deficient state due to pre-existing disease and malnutrition, vitamin D metabolism is dysregulated in critical illness [38] and concentrations fall rapidly after ICU admission [39]. Furthermore, vitamin D insufficiency/deficiency has been associated with a range of poor outcomes in critical illness [37,40–42]. Vitamin D insufficiency leads to secondary hyperparathyroidism and a concentration of 50 nmol/L total 25(OH)D is required for optimum PTH concentrations [43]. 87% of the critical illness survivors had total 25(OH)D <50 nmol/L, which would be associated with secondary hyperparathyroidism and the potential for associated loss of bone mineral density. Critical illness survivors suffer accelerated loss of bone mineral density in the year after ICU discharge (compared to matched controls) and increased 10-year fracture risk [13]. Our findings implicate vitamin D insufficiency in this process.

There is evidence that vitamin D supplementation can improve circulating total 25(OH)D concentrations in critically ill patients [34,35,44], but evidence of a beneficial effect on outcomes is

For peer review only - http://bmjopen.bmj.com/site/about/guidelines.xhtml

less clear. High-dose vitamin D supplementation in COVID-19 [12] and critical illness [44] has been shown to increase plasma 25(OH)D concentrations 7-days post-supplementation but no significant reduction in the length of hospital stay or acute outcomes including in-hospital mortality, admission to ICU or requirement for IMV were demonstrated [12,44,45]. Longer term outcomes such as bone health have not been evaluated. Conversely, a report of an open-label randomized trial in COVID-19 patients showed that those who were given high-dose 25(OH)D3 (instead of vitamin D3 as in the above mentioned studies) on admission and then subsequent doses on days 3, 7 and then weekly, were less likely to require ICU admission [46]. We identified that vitamin D insufficiency was present early in the course of COVID-19 and influenza A (10 and 7 days after symptom onset respectively) indicating that timing of supplementation may be an important factor when designing future supplementation studies. We propose that future studies examining effects on disease progression should investigate the effects of vitamin D supplementation given earlier in the course of disease, closer to symptom onset rather than after hospitalisation. The longer-term effects of persistent vitamin D insufficiency/deficiency in survivors of critical illness also requires further investigation especially in the context of bone health which could be independently evaluated using sequential measurement of bone turnover markers and serum PTH.

In conclusion, vitamin D deficiency/insufficiency was present in the majority of hospitalised patients with COVID-19 or influenza A and scaled with severity, highlighting that reduced concentrations of vitamin D is common to these disease states and distinct patient cohorts. For the first time, free and total 25(OH)D were studied in COVID-19 demonstrating consistent results. It is not clear whether vitamin D status led to poor clinical outcome or was a consequence of illness severity. Randomised trials will be necessary to determine whether a causal relationship exists between vitamin D early in the course of disease and development of critical illness. Since vitamin D deficiency/insufficiency persisted at concentrations expected to disrupt bone metabolism in critical illness survivors, investigation of longer-term bone health outcomes is also warranted.

For peer review only

ACKNOWLEDGEMENTS

The ISARIC4C work uses data provided by patients and collected by the NHS as part of their care and support. We are extremely grateful to the 2648 front-line NHS clinical and research staff and volunteer medical students who collected this data in challenging circumstances; and the generosity of the participants and their families for their individual contributions in these difficult times. Thanks to Joanna Simpson and Patricia Lee who were involved in sample management and free 25(OH)D data collection during the analytical process. Thanks to Catherine Duff and Sharon Hannah who assisted in the health and safety aspects of the project, and to the Clinical Biochemistry Department at Imperial College Healthcare NHS Trust for analysing the samples from influenza patients in 2011. We also acknowledge the support of Jeremy J Farrar (Wellcome Trust) and Nahoko Shindo (WHO).

COMPETING INTERESTS STATEMENT

RJM and EH are part of the VitDAL which provides a 25(OH)D assay service on a not-for-profit basis.

FUNDING

This work was supported by grants from: the National Institute for Health Research (NIHR) [award CO-CIN-01], the Medical Research Council [grant MC_PC_19059] and by the NIHR Health Protection Research Unit (HPRU) in Emerging and Zoonotic Infections at University of Liverpool in partnership with Public Health England (PHE), in collaboration with Liverpool School of Tropical Medicine and the University of Oxford [award 200907], NIHR HPRU in Respiratory Infections at Imperial College London with PHE [award 200927], Wellcome Trust and Department for International Development [215091/Z/18/Z], and the Bill and Melinda Gates Foundation [OPP1209135], and Liverpool Experimental Cancer Medicine Centre (Grant Reference: C18616/A25153), NIHR Biomedical Research Centre at Imperial College London [IS-BRC-1215-20013], EU Platform for European Preparedness Against (Re-) emerging Epidemics (PREPARE) [FP7 project 602525] and NIHR Clinical Research Network for providing infrastructure support for this research. LT is supported by a Wellcome Trust fellowship

For peer review only

For peer review only - http://bmjopen.bmj.com/site/about/guidelines.xhtml

[205228/Z/16/Z]. The MOSAIC study was supported by the Wellcome Trust (087805/Z/08/Z) and the Medical Research Council HIC-Vac network (MR/R005982/1). PJMO is supported by a NIHR Senior Investigator Award [award 201385]. For the purpose of Open Access, the authors have applied a CC BY public copyright licence to any Author Accepted Manuscript version arising from this submission. RJS is funded by a EMINENT GSK-NIHR research training fellowship supported by the Cambridge NIHR Biomedical Research Centre. The RECOVER trial was funded by the Chief Scientists Office Scotland (CZH/4/53), trial registration ISRCTN09412438. Professor Shankar-Hari is funded by the National Institute for Health Research Clinician Scientist Award (CS-2016-16-011). We acknowledge the financial support of NHS Research Scotland (NRS), for the Mass Spectrometry Core, Edinburgh Clinical Research Facility.

The views expressed in this publication are those of the author(s) and not necessarily those of the NHS, the National Institute for Health Research or the Department of Health and Social Care, or other funders.

For peer review only

DATA SHARING

Access to all data and samples collected by ISARIC4C are controlled by an Independent Data and Materials Access Committee composed of representatives of research funders, academia, clinical medicine, public health, and industry. The application process for access to the data is available on the ISARIC4C website (https://isaric4c.net).

For peer review only - http://bmjopen.bmj.com/site/about/guidelines.xhtml

AUTHOR CONTRIBUTIONS

Conceptualisation: EA Hurst, RJ Mellanby, CD Russell, L Turtle, PJM Openshaw, JK Baillie and MG Semple

Data curation: EA Hurst, DM Griffith, J Dunning and CD Russell

Formal analysis: EA Hurst, I Handel and CD Russell

Funding acquisition: RJ Mellanby, PJM Openshaw, JK Baillie and MG Semple

Investigation: EA Hurst, RJ Mellanby, I Handel, DM Griffith, AG Rossi, TS Walsh, M Shankar-Hari, NZ Homer, SG Denham, K Devine, PA Holloway, RS Thwaites, RJ Samanta, J Dunning, C Summers, L Turtle, PJM Openshaw, JK Baillie, MG Semple and CD Russell

Project administration: SC Moore, HE Hardwick and W Oosthuyzen

Data visualisation: I Handel and CD Russell

Supervision: RJ Mellanby, MG Semple, PJM Openshaw, JK Baillie and CD Russell

Writing original draft: EA Hurst and CD Russell

Writing review & editing: EA Hurst, RJ Mellanby, I Handel, DM Griffith, AG Rossi, TS Walsh, M Shankar-Hari, NZ Homer, SG Denham, K Devine, PA Holloway, RS Thwaites, RJ Samanta, J Dunning, C Summers, L Turtle, PJM Openshaw, JK Baillie, MG Semple and CD Russell

Validation: EA Hurst, RJ Mellanby and CD Russell

For peer review only

For peer review only - http://bmjopen.bmj.com/site/about/guidelines.xhtml

AUTHORSHIP SUPPLEMENT

ISARIC4C Investigators

Consortium Lead Investigator: J Kenneth Baillie. Chief Investigator: Malcolm G Semple. Co-Lead Investigator: Peter JM Openshaw. ISARIC Clinical Coordinator: Gail Carson. Co-Investigator: Beatrice Alex, Benjamin Bach, Wendy S Barclay, Debby Bogaert, Meera Chand, Graham S Cooke, Annemarie B Docherty, Jake Dunning, Ana da Silva Filipe, Tom Fletcher, Christopher A Green, Ewen M Harrison, Julian A Hiscox, Antonia Ying Wai Ho, Peter W Horby, Samreen Ijaz, Saye Khoo, Paul Klenerman, Andrew Law, Wei Shen Lim, Alexander J Mentzer, Laura Merson, Alison M Meynert, Mahdad Noursadeghi, Shona C Moore, Massimo Palmarini, William A Paxton, Georgios Pollakis, Nicholas Price, Andrew Rambaut, David L Robertson, Clark D Russell, Vanessa Sancho-Shimizu, Janet T Scott, Thushan de Silva, Louise Sigfrid, Tom Solomon, Shiranee Sriskandan, David Stuart, Charlotte Summers, Richard S Tedder, Emma C Thomson, AA Roger Thompson, Ryan S Thwaites, Lance CW Turtle, Rishi K Gupta, Maria Zambon. Project Manager: Hayley Hardwick, Chloe Donohue, Ruth Lyons, Fiona Griffiths, Wilna Oosthuyzen. Data Analyst: Lisa Norman, Riinu Pius, Thomas M Drake, Cameron J Fairfield, Stephen R Knight, Kenneth A Mclean, Derek Murphy, Catherine A Shaw. Data and Information System Manager: Jo Dalton, Michelle Girvan, Egle Saviciute, Stephanie Roberts, Janet Harrison, Laura Marsh, Marie Connor, Sophie Halpin, Clare Jackson, Carrol Gamble. Data Integration and Presentation: Gary Leeming, Andrew Law, Murray Wham, Sara Clohisey, Ross Hendry, James Scott-Brown. Material Management: William Greenhalf, Victoria Shaw, Sara McDonald. Patient Engagement: Seán Keating. Outbreak Laboratory Staff and Volunteers: Katie A. Ahmed, Jane A Armstrong, Milton Ashworth, Innocent G Asiimwe, Siddharth Bakshi, Samantha L Barlow, Laura Booth, Benjamin Brennan, Katie Bullock, Benjamin WA Catterall, Jordan J Clark, Emily A Clarke, Sarah Cole, Louise Cooper, Helen Cox, Christopher Davis, Oslem Dincarslan, Chris Dunn, Philip Dyer, Angela Elliott, Anthony Evans, Lorna Finch, Lewis WS Fisher, Terry Foster, Isabel Garcia-Dorival, William Greenhalf, Philip Gunning, Catherine Hartley, Rebecca L Jensen, Christopher B Jones, Trevor R Jones, Shadia Khandaker, Katharine King, Robyn T. Kiy, Chrysa Koukorava, Annette Lake, Suzannah Lant, Diane Latawiec, Lara

For peer review only

Lavelle-Langham, Daniella Lefteri, Lauren Lett, Lucia A Livoti, Maria Mancini, Sarah McDonald, Laurence McEvoy, John McLauchlan, Soeren Metelmann, Nahida S Miah, Joanna Middleton, Joyce Mitchell, Shona C Moore, Ellen G Murphy, Rebekah Penrice-Randal, Jack Pilgrim, Tessa Prince, Will Reynolds, P. Matthew Ridley, Debby Sales, Victoria E Shaw, Rebecca K Shears, Benjamin Small, Krishanthi S Subramaniam, Agnieska Szemiel, Aislynn Taggart, Jolanta Tanianis-Hughes, Jordan Thomas, Erwan Trochu, Libby van Tonder, Eve Wilcock, J. Eunice Zhang, Lisa Flaherty, Nicole Maziere, Emily Cass, Alejandra Doce Carracedo, Nicola Carlucci, Anthony Holmes, Hannah Massey. Edinburgh Laboratory Staff and Volunteers: Lee Murphy, Nicola Wrobel, Sarah McCafferty, Kirstie Morrice, Alan MacLean. Local Principal Investigators: Kayode Adeniji, Daniel Agranoff, Ken Agwuh, Dhiraj Ail, Erin L. Aldera, Ana Alegria, Brian Angus, Abdul Ashish, Dougal Atkinson, Shahedal Bari, Gavin Barlow, Stella Barnass, Nicholas Barrett, Christopher Bassford, Sneha Basude, David Baxter, Michael Beadsworth, Jolanta Bernatoniene, John Berridge, Nicola Best, Pieter Bothma, David Chadwick, Robin Brittain-Long, Naomi Bulteel, Tom Burden, Andrew Burtenshaw, Vikki Caruth, David Chadwick, Duncan Chambler, Nigel Chee, Jenny Child, Srikanth Chukkambotla, Tom Clark, Paul Collini, Catherine Cosgrove, Jason Cupitt, Maria-Teresa Cutino-Moguel, Paul Dark, Chris Dawson, Samir Dervisevic, Phil Donnison, Sam Douthwaite, Ingrid DuRand, Ahilanadan Dushianthan, Tristan Dyer, Cariad Evans, Chi Eziefula, Chrisopher Fegan, Adam Finn, Duncan Fullerton, Sanjeev Garg, Sanjeev Garg, Atul Garg, Effrossyni Gkrania-Klotsas, Jo Godden, Arthur Goldsmith, Clive Graham, Elaine Hardy, Stuart Hartshorn, Daniel Harvey, Peter Havalda, Daniel B Hawcutt, Maria Hobrok, Luke Hodgson, Anil Hormis, Michael Jacobs, Susan Jain, Paul Jennings, Agilan Kaliappan, Vidya Kasipandian, Stephen Kegg, Michael Kelsey, Jason Kendall, Caroline Kerrison, Ian Kerslake, Oliver Koch, Gouri Koduri, George Koshy, Shondipon Laha, Steven Laird, Susan Larkin, Tamas Leiner, Patrick Lillie, James Limb, Vanessa Linnett, Jeff Little, Mark Lyttle, Michael MacMahon, Emily MacNaughton, Ravish Mankregod, Huw Masson, Elijah Matovu, Katherine McCullough, Ruth McEwen, Manjula Meda, Gary Mills, Jane Minton, Mariyam Mirfenderesky, Kavya Mohandas, Quen Mok, James Moon, Elinoor Moore, Patrick Morgan, Craig Morris, Katherine Mortimore, Samuel Moses, Mbiye Mpenge, Rohinton Mulla, Michael Murphy,

For peer review only

Megan Nagel, Thapas Nagarajan, Mark Nelson, Matthew K. O'Shea, Igor Otahal, Marlies Ostermann, Mark Pais, Carlo Palmieri, Selva Panchatsharam, Danai Papakonstantinou, Hassan Paraiso, Brij Patel, Natalie Pattison, Justin Pepperell, Mark Peters, Mandeep Phull, Stefania Pintus, Jagtur Singh Pooni, Frank Post, David Price, Rachel Prout, Nikolas Rae, Henrik Reschreiter, Tim Reynolds, Neil Richardson, Mark Roberts, Devender Roberts, Alistair Rose, Guy Rousseau, Brendan Ryan, Taranprit Saluja, Aarti Shah, Prad Shanmuga, Anil Sharma, Anna Shawcross, Jeremy Sizer, Manu Shankar-Hari, Richard Smith, Catherine Snelson, Nick Spittle, Nikki Staines, Tom Stambach, Richard Stewart, Pradeep Subudhi, Tamas Szakmany, Kate Tatham, Jo Thomas, Chris Thompson, Robert Thompson, Ascanio Tridente, Darell Tupper-Carey, Mary Twagira, Andrew Ustianowski, Nick Vallotton, Lisa Vincent-Smith, Shico Visuvanathan, Alan Vuylsteke, Sam Waddy, Rachel Wake, Andrew Walden, Ingeborg Welters, Tony Whitehouse, Paul Whittaker, Ashley Whittington, Padmasayee Papineni, Meme Wijesinghe, Martin Williams, Lawrence Wilson, Sarah Cole, Stephen Winchester, Martin Wiselka, Adam Wolverson, Daniel G Wootton, Andrew Workman, Bryan Yates, Peter Young.

For peer review only

For peer review only - http://bmjopen.bmj.com/site/about/guidelines.xhtml

For peer review only

For peer review only - http://bmjopen.bmj.com/site/about/guidelines.xhtml

FIGURE CAPTIONS

Figure 1: Total 25(OH)D in COVID-19, influenza A and survivors of critical illness

Violin plots of total 25(OH)D concentrations (nmol/L). The solid line within the plot represents the median and the dashed lines represent the interquartile range. The dotted lines on the y-axis represent the thresholds for total vitamin D insufficiency (25-50nmol/L) and deficiency (<25nmol/L). Patients with (A) COVID-19 (n=295) and (B) influenza A (from 2009 H1N1 pandemic, n=93) are stratified by receipt of invasive mechanical ventilation. Groups are compared by Mann-Whitney test. The stacked bar charts represent the proportion of patients in each sub-group with sufficient (green), insufficient (orange), or deficient (red) total vitamin D status, compared by Chi-squared test. (C) Non-selected critical illness survivors (n=139, recruited prior to the COVID-19 pandemic) at the time of ICU discharge.

Figure 2: Total and free 25(OH)D and outcomes in COVID-19 and influenza A

Smoothed predicted probability of outcomes (invasive mechanical ventilation or in-hospital mortality) vs. total or free 25(OH)D concentration (with other co-variates at mean values) from the binary logistic regression multivariable models. Grey ribbon represents estimated 95% confidence interval and the x-axis ticks show observations.

Figure 3: Free 25(OH)D in COVID-19

(A) Simple linear regression line and 95% confidence interval (dashed lines) representing the correlation between total and free 25(OH)D concentrations in COVID-19. (B) Violin plot of free 25(OH)D concentrations (pg/ml) in patients with COVID-19 stratified by receipt of invasive mechanical ventilation. The solid line within the plot represents the median and the dashed lines represent the interquartile range. Groups are compared by Mann-Whitney test.

For peer review only - http://bmjopen.bmj.com/site/about/guidelines.xhtml

REFERENCES

1. Hewison M. Vitamin D and the intracrinology of innate immunity. Mol Cell Endocrinol **2010**; 321:103–111. Available at: http://www.sciencedirect.com/science/article/pii/S0303720710000894.

2. Chun RF, Lauridsen AL, Suon L, et al. Vitamin D-Binding Protein Directs Monocyte Responses to 25-Hydroxy- and 1,25-Dihydroxyvitamin D. J Clin Endocrinol Metab **2010**; 95:3368–3376. Available at: https://doi.org/10.1210/jc.2010-0195.

3. Sabetta JR, DePetrillo P, Cipriani RJ, Smardin J, Burns LA, Landry ML. Serum 25-Hydroxyvitamin D and the Incidence of Acute Viral Respiratory Tract Infections in Healthy Adults. PLoS One **2010**; 5:e11088. Available at: https://doi.org/10.1371/journal.pone.0011088.

4. Monlezun D, Bittner E, Christopher K, Camargo C, Quraishi S. Vitamin D Status and Acute Respiratory Infection: Cross Sectional Results from the United States National Health and Nutrition Examination Survey, 2001–2006. Nutrients **2015**; 7:1933–1944. Available at: http://www.mdpi.com/2072-6643/7/3/1933. Accessed 15 December 2020.

5 Dancer RCA, Parekh D, Lax S, et al. Vitamin D deficiency contributes directly to the acute respiratory distress syndrome (ARDS). Thorax **2015**; 70:617 LP – 624. Available at: http://thorax.bmj.com/content/70/7/617.abstract.

6. Meltzer DO, Best TJ, Zhang H, Vokes T, Arora V, Solway J. Association of Vitamin D Status and Other Clinical Characteristics With COVID-19 Test Results. JAMA Netw Open **2020**; 3:e2019722–e2019722. Available at: https://doi.org/10.1001/jamanetworkopen.2020.19722.

7. Kaufman HW, Niles JK, Kroll MH, Bi C, Holick MF. SARS-CoV-2 positivity rates associated with circulating 25-hydroxyvitamin D levels. PLoS One **2020**; 15:e0239252. Available at: https://doi.org/10.1371/journal.pone.0239252.

8. Marik PE, Kory P, Varon J. Does vitamin D status impact mortality from SARS-CoV-2 infection? Med Drug Discov **2020**; 6:100041. Available at:

For peer review only

http://www.sciencedirect.com/science/article/pii/S2590098620300282.

9. Carolien Mathyssen, Ghislaine Gayan-Ramirez, Roger Bouillon WJ. Vitamin D supplementation in respiratory diseases: evidence from randomized controlled trials. Polish Arch Intern Med **2017**; 127:775–784.

10. Martineau AR, Jolliffe DA, Greenberg L, et al. Vitamin D supplementation to prevent acute respiratory infections: individual participant data meta-analysis. 2019; 23:2. Available at: https://doi.org/10.3310/hta23020.

11. Ganmaa D, Uyanga B, Zhou X, et al. Vitamin D Supplements for Prevention of Tuberculosis Infection and Disease. N Engl J Med **2020**; 383:359–368. Available at: https://doi.org/10.1056/NEJMoa1915176.

12. Murai IH, Fernandes AL, Sales LP, et al. Effect of a Single High Dose of Vitamin D3 on Hospital Length of Stay in Patients With Moderate to Severe COVID-19: A Randomized Clinical Trial. JAMA **2021**; 325:1053–1060. Available at: https://doi.org/10.1001/jama.2020.26848.

13. Orford NR, Lane SE, Bailey M, et al. Changes in Bone Mineral Density in the Year after Critical Illness. Am J Respir Crit Care Med **2015**; 193:736–744. Available at: https://doi.org/10.1164/rccm.201508-1514OC.

14. Docherty AB, Harrison EM, Green CA, et al. Features of 20 133 UK patients in hospital with covid-19 using the ISARIC WHO Clinical Characterisation Protocol: prospective observational cohort study. BMJ **2020**; 369:m1985. Available at: http://www.bmj.com/content/369/bmj.m1985.abstract.

15. Walsh TS, Salisbury LG, Merriweather JL, et al. Increased hospital-based physical rehabilitation and information provision after intensive care unit discharge: The RECOVER randomized clinical trial. JAMA Intern Med **2015**; 175.

16. Griffith DM, Lewis S, Rossi AG, et al. Systemic inflammation after critical illness: relationship with physical recovery and exploration of potential mechanisms. Thorax **2016**; 71:820 LP – 829. Available at: http://thorax.bmj.com/content/71/9/820.abstract.

For peer review only

17. Dunning J, Blankley S, Hoang LT, et al. Progression of whole-blood transcriptional signatures from interferon-induced to neutrophil-associated patterns in severe influenza. Nat Immunol **2018**; 19:625–635. Available at: https://doi.org/10.1038/s41590-018-0111-5.

18. Hurst EA, Homer NZ, Gow AG, et al. Vitamin D status is seasonally stable in northern European dogs. Vet Clin Pathol **2020**; n/a. Available at: https://doi.org/10.1111/vcp.12859.

19. Sai AJ, Walters RW, Fang X, Gallagher JC. Relationship between Vitamin D, Parathyroid Hormone, and Bone Health. J Clin Endocrinol Metab **2011**; 96:E436–E446. Available at: https://doi.org/10.1210/jc.2010-1886.

20. Wood SN. Thin plate regression splines. J R Stat Soc Ser B **2003**; 65:95–114. Available at: https://econpapers.repec.org/RePEc:bla:jorssb:v:65:y:2003:i:1:p:95-114.

21. Wood SN. Fast stable restricted maximum likelihood and marginal likelihood estimation of semiparametric generalized linear models. J R Stat Soc Ser B (Statistical Methodol **2011**; 73:3–36. Available at: https://doi.org/10.1111/j.1467-9868.2010.00749.x.

22. Lubin JH, Colt JS, Camann D, et al. Epidemiologic Evaluation of Measurement Data in the Presence of Detection Limits. Environ Health Perspect **2004**; 112:1691–1696. Available at: https://doi.org/10.1289/ehp.7199.

23. R Foundation for Statistical Computing. R Core Team (2020). R: A language and environment for statistical computing. 2020;

24. Thwaites RS, Sanchez Sevilla Uruchurtu A, Siggins MK, et al. Inflammatory profiles across the spectrum of disease reveal a distinct role for GM-CSF in severe COVID-19. Sci Immunol **2021**; 6:eabg9873. Available at: http://immunology.sciencemag.org/content/6/57/eabg9873.abstract.

25. D'Avolio A, Avataneo V, Manca A, et al. 25-Hydroxyvitamin D Concentrations Are Lower in Patients with Positive PCR for SARS-CoV-2. Nutrients **2020**; 12:1359. Available at: https://www.mdpi.com/2072-6643/12/5/1359. Accessed 14 December 2020.

26. Panagiotou G, Tee SA, Ihsan Y, et al. Low serum 25-hydroxyvitamin D (25[OH]D) levels in

For peer review only

For peer review only - http://bmjopen.bmj.com/site/about/guidelines.xhtml

patients hospitalized with COVID-19 are associated with greater disease severity. Clin Endocrinol (Oxf) **2020**; 93:508–511. Available at: https://doi.org/10.1111/cen.14276.

27. Carpagnano GE, Di Lecce V, Quaranta VN, et al. Vitamin D deficiency as a predictor of poor prognosis in patients with acute respiratory failure due to COVID-19. J Endocrinol Invest **2020**; Available at: https://doi.org/10.1007/s40618-020-01370-x.

28. Yuk J-M, Shin D-M, Lee H-M, et al. Vitamin D3 Induces Autophagy in Human Monocytes/Macrophages via Cathelicidin. Cell Host Microbe **2009**; 6:231–243. Available at: https://doi.org/10.1016/j.chom.2009.08.004.

29. Liu PT, Stenger S, Li H, et al. Toll-Like Receptor Triggering of a Vitamin D-Mediated Human Antimicrobial Response. Science (80-) **2006**; 311:1770 LP – 1773. Available at: http://science.sciencemag.org/content/311/5768/1770.abstract.

30. Campbell GR, Spector SA. Hormonally Active Vitamin D3 (1α,25-Dihydroxycholecalciferol) Triggers Autophagy in Human Macrophages That Inhibits HIV-1 Infection. J Biol Chem **2011**; 286:18890–18902. Available at: http://www.jbc.org/content/286/21/18890.abstract.

31. Khare D, Godbole NM, Pawar SD, et al. Calcitriol [1, 25[OH]2 D3] pre- and post-treatment suppresses inflammatory response to influenza A (H1N1) infection in human lung A549 epithelial cells. Eur J Nutr **2013**; 52:1405–1415. Available at: https://doi.org/10.1007/s00394-012-0449-7.

32. Chun RF, Peercy BE, Adams JS, Hewison M. Vitamin D Binding Protein and Monocyte Response to 25-Hydroxyvitamin D and 1,25-Dihydroxyvitamin D: Analysis by Mathematical Modeling. PLoS One **2012**; 7:e30773. Available at: https://doi.org/10.1371/journal.pone.0030773.

33. Larner DP, Jenkinson C, Chun RF, Westgate CSJ, Adams JS, Hewison M. Free versus total serum 25-hydroxyvitamin D in a murine model of colitis. J Steroid Biochem Mol Biol **2019**; 189:204–209. Available at: http://www.sciencedirect.com/science/article/pii/S0960076018304084.

For peer review only - http://bmjopen.bmj.com/site/about/guidelines.xhtml

34. Han JE, Alvarez JA, Jones JL, et al. Impact of high-dose vitamin D3 on plasma free 25-hydroxyvitamin D concentrations and antimicrobial peptides in critically ill mechanically ventilated adults. Nutrition **2017**; 38:102–108. Available at: http://www.sciencedirect.com/science/article/pii/S0899900717300345.

35. Quraishi SA, De Pascale G, Needleman JS, et al. Effect of Cholecalciferol Supplementation on Vitamin D Status and Cathelicidin Levels in Sepsis: A Randomized, Placebo-Controlled Trial. Crit Care Med **2015**; 43. Available at: https://journals.lww.com/ccmjournal/Fulltext/2015/09000/Effect_of_Cholecalciferol_Supplementation_on.18.aspx.

36. Braun A, Chang D, Mahadevappa K, et al. Association of low serum 25-hydroxyvitamin D levels and mortality in the critically ill*. Crit Care Med **2011**; 39. Available at: https://journals.lww.com/ccmjournal/Fulltext/2011/04000/Association_of_low_serum_25_hydroxyvitamin_D.9.aspx.

37. Lucidarme O, Messai E, Mazzoni T, Arcade M, du Cheyron D. Incidence and risk factors of vitamin D deficiency in critically ill patients: results from a prospective observational study. Intensive Care Med **2010**; 36:1609–1611. Available at: https://doi.org/10.1007/s00134-010-1875-8.

38. Czarnik T, Czarnik A, Gawda R, et al. Vitamin D kinetics in the acute phase of critical illness: A prospective observational study. J Crit Care **2018**; 43:294–299. Available at: http://www.sciencedirect.com/science/article/pii/S0883944117305312.

39. Amrein K, Christopher KB, McNally JD. Understanding vitamin D deficiency in intensive care patients. Intensive Care Med **2015**; 41:1961–1964. Available at: https://doi.org/10.1007/s00134-015-3937-4.

40. Braun AB, Litonjua AA, Moromizato T, Gibbons FK, Giovannucci E, Christopher KB. Association of low serum 25-hydroxyvitamin D levels and acute kidney injury in the critically ill*. Crit Care Med **2012**; 40. Available at:

For peer review only - http://bmjopen.bmj.com/site/about/guidelines.xhtml

https://journals.lww.com/ccmjournal/Fulltext/2012/12000/Association_of_low_serum_25_hydroxyvitamin_D.8.aspx.

41. Arnson Y, Gringauz I, Itzhaky D, Amital H. Vitamin D deficiency is associated with poor outcomes and increased mortality in severely ill patients. QJM An Int J Med **2012**; 105:633–639. Available at: https://doi.org/10.1093/qjmed/hcs014.

42. de Haan K, Groeneveld ABJ, de Geus HRH, Egal M, Struijs A. Vitamin D deficiency as a risk factor for infection, sepsis and mortality in the critically ill: systematic review and meta-analysis. Crit Care **2014**; 18:660. Available at: https://doi.org/10.1186/s13054-014-0660-4.

43. Malabanan A, Veronikis IE, Holick MF. Redefining vitamin D insufficiency. Lancet **1998**; 351:805–806. Available at: https://doi.org/10.1016/S0140-6736(05)78933-9.

44. Amrein K, Schnedl C, Holl A, et al. Effect of High-Dose Vitamin D3 on Hospital Length of Stay in Critically Ill Patients With Vitamin D Deficiency: The VITdAL-ICU Randomized Clinical Trial. JAMA **2014**; 312:1520–1530. Available at: https://doi.org/10.1001/jama.2014.13204.

45. National Heart, Lung, and Blood Institute PETAL Clinical Trials Network, Ginde AA, Brower RG, Caterino JM, Finck L, Banner-Goodspeed VM, Grissom CK, Hayden D, Hough CL, Hyzy RC, Khan A, Levitt JE, Park PK, Ringwood N, Rivers EP, Self WH, Shapiro NI, Thomp TD. Early High-Dose Vitamin D3 for Critically Ill, Vitamin D–Deficient Patients. N Engl J Med **2019**; 381:2529–2540. Available at: https://doi.org/10.1056/NEJMoa1911124.

46. Entrenas Castillo M, Entrenas Costa LM, Vaquero Barrios JM, et al. "Effect of calcifediol treatment and best available therapy versus best available therapy on intensive care unit admission and mortality among patients hospitalized for COVID-19: A pilot randomized clinical study". J Steroid Biochem Mol Biol **2020**; 203:105751. Available at: https://www.sciencedirect.com/science/article/pii/S0960076020302764.

For peer review only

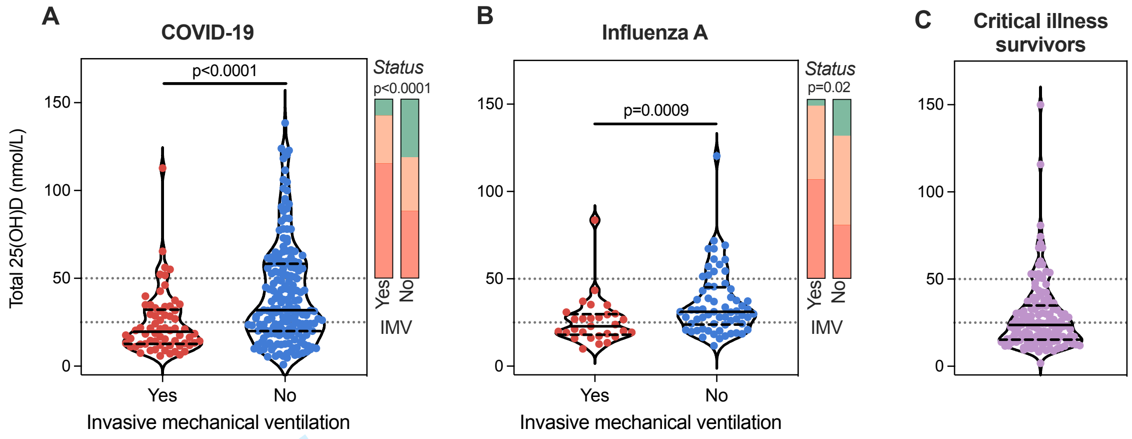

Figure 1: Total 25(OH)D in COVID-19, influenza A and survivors of critical illness

Violin plots of total 25(OH)D concentrations (nmol/L). The solid line within the plot represents the median and the dashed lines represent the interquartile range. The dotted lines on the y-axis represent the thresholds for total vitamin D insufficiency (25-50nmol/L) and deficiency (<25nmol/L). Patients with **(A)** COVID-19 (n=295) and **(B)** influenza A (from 2009 H1N1 pandemic, n=93) are stratified by receipt of invasive mechanical ventilation. Groups are compared by Mann-Whitney test. The stacked bar charts represent the proportion of patients in each sub-group with sufficient (green), insufficient (orange), or deficient (red) total vitamin D status, compared by Chi-squared test. **(C)** Non-selected critical illness survivors (n=139, recruited prior to the COVID-19 pandemic) at the time of ICU discharge.

For peer review only

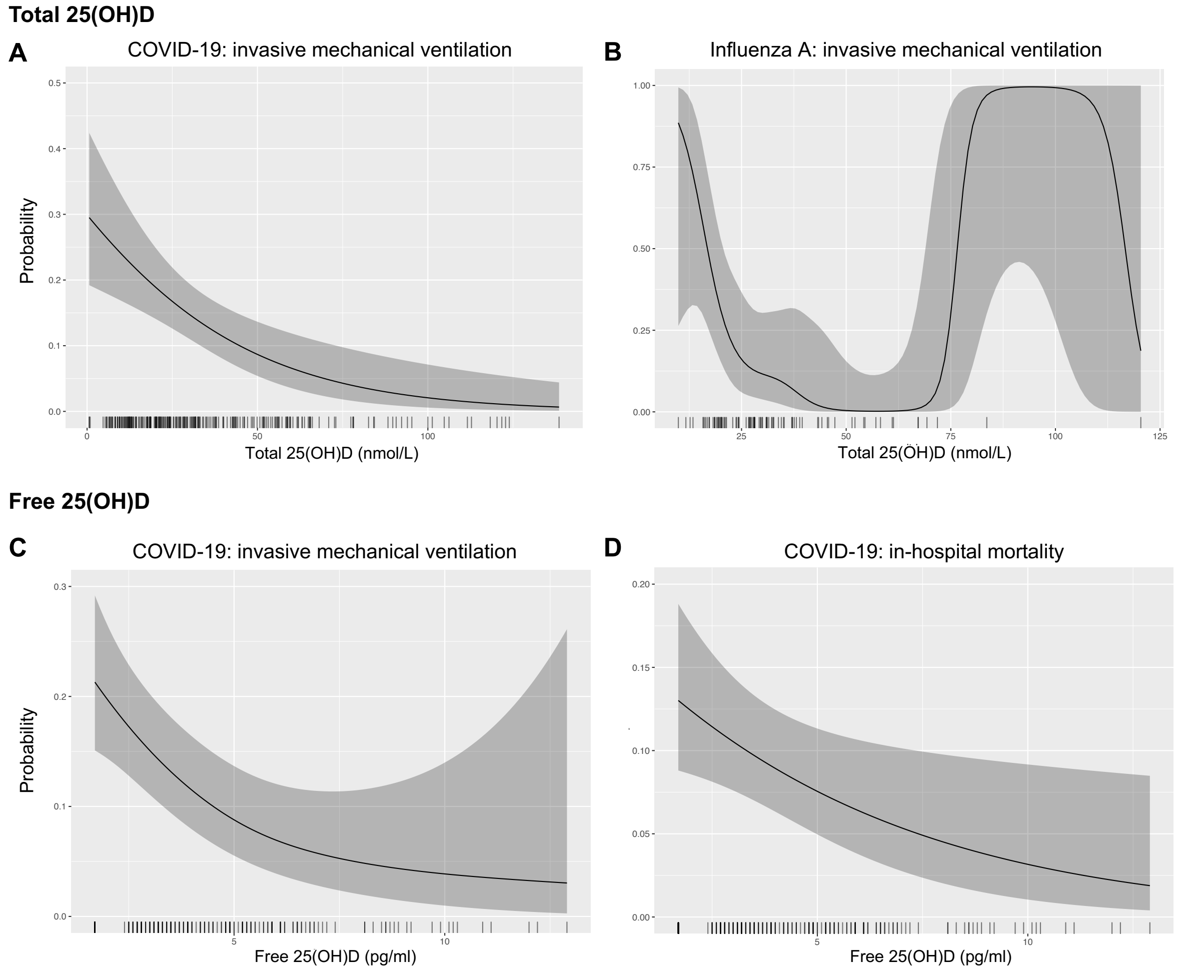

Figure 2: Total and free 25(OH)D and outcomes in COVID-19 and influenza A

Smoothed predicted probability of outcomes (invasive mechanical ventilation or in-hospital mortality) vs. total or free 25(OH)D concentration (with other co-variates at mean values) from the binary logistic regression multivariable models. Grey ribbon represents estimated 95% confidence interval and the x-axis ticks show observations.

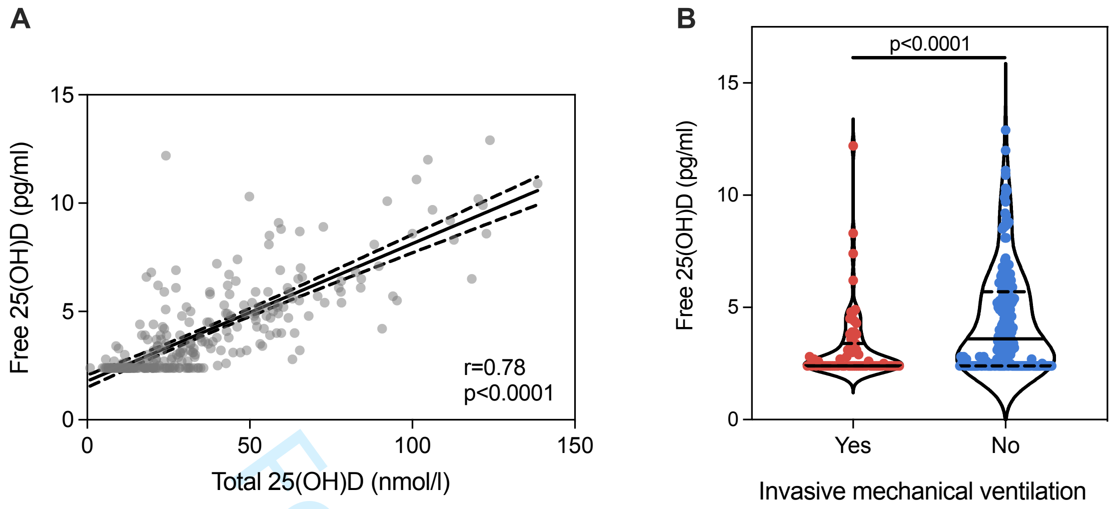

Figure 3: Free 25(OH)D in COVID-19

(A) Simple linear regression line and 95% confidence interval (dashed lines) representing the correlation between total and free 25(OH)D concentrations in COVID-19. **(B)** Violin plot of free 25(OH)D concentrations (pg/ml) in patients with COVID-19 stratified by receipt of invasive mechanical ventilation. The solid line within the plot represents the median and the dashed lines represent the interquartile range. Groups are compared by Mann-Whitney test.

For peer review only

SUPPLEMENTARY DATA

Supplementary Table 1: LC-MS/MS method parameters

**Parameter**	**COVID-19** (n=259) **/ ICU** (n=139) **sample method**	**Influenza A** (n=93) **/ healthy controls** (n=36) **sample method**
**Sample preparation**		
Isotopically labelled internal standards	d_3-25(OH)D2<br>$^{13}C_5$-25(OH)D3	-<br>d_6-25(OH)D3
Extraction method	Automated SLE	PPT + LLE
Derivatization	DMEQ-TAD	-
**LC-MS instrumentation**		
LC-MS system	Shimadzu Nexera UPLC – Sciex QTrap 6500+	Waters ACQUITY TQD UPLC/MS/MS
LC column	Raptor Fluorophenyl column (2.7µm 100 Å, 100 x 2.1 mm)	Phenyl reversed phase LC column
Ionization mode	ESI, positive	Turbulon Spray, positive
Detection mode	MRM	MRM
**Method specifications**		
LLOQ	0.5 nmol/L 25(OH)D2<br>4 nmol/L 25(OH)D3	10 nmol/L 25(OH)D2<br>10 nmol/L 25(OH)D3
Inter-assay precision (CV%)	<11.5% 25(OH)D2<br><11.5% 25(OH)D3	<11% 25(OH)D2<br><10% 25(OH)D3

n – number of patient samples analysed; d – deuterium labelled; ^{13}C – carbon 13 labelled; SLE – supported liquid extraction performed on the Biotage® Extrahera™; PPT – protein precipitation; LLE – liquid liquid extraction using n-hexane; ESI – electrospray ionization; MRM – multiple reaction monitoring; LLOQ – lower limit of quantification.

For peer review only

Supplementary Table 2: Multivariable analyses of total 25(OH)D and vitamin D status and outcomes in COVID-19

Variable	Odds ratio	p-value
**Vitamin D status**		
Receipt of IMV		
**Sufficient**[a]	0.26 (0.1-0.62)	**0.004**
Male sex	2.41 (1.18-4.91)	**0.015**
Comorbidity count[b]	-	0.805
Day of illness[b]	-	0.462
Age[b]	-	**0.018**
In-hospital mortality		
**Sufficient**[a]	0.27 (0.11-0.68)	**0.005**
Male sex	2.52 (1.13-5.63)	**0.024**
Comorbidity count[b]		**0.016**
Day of illness[b]	-	0.576
Age[b]	-	0.059
**Total 25(OH)D concentration**		
In-hospital mortality		
Male sex	2.49 (1.12-5.57)	**0.026**
Comorbidity count[b]	-	**0.030**
**Total 25(OH)D**[b]	-	0.068
Day of illness[b]	-	0.495
Age[b]	-	**0.046**

[a]total 25(OH)D >50 nmol/l
[b]smoothed

Supplementary Table 3: Multivariable analysis of total 25(OH)D concentration and in-hospital mortality in influenza A

Variable	Odds ratio	p-value
In-hospital mortality		
Male sex	0.84	0.798
Comorbidity count[a]	-	0.539
**Total 25(OH)D**[a]	-	0.421
Day of illness[a]	-	0.244
Age[a]	-	0.200

[a]smoothed

For peer review only

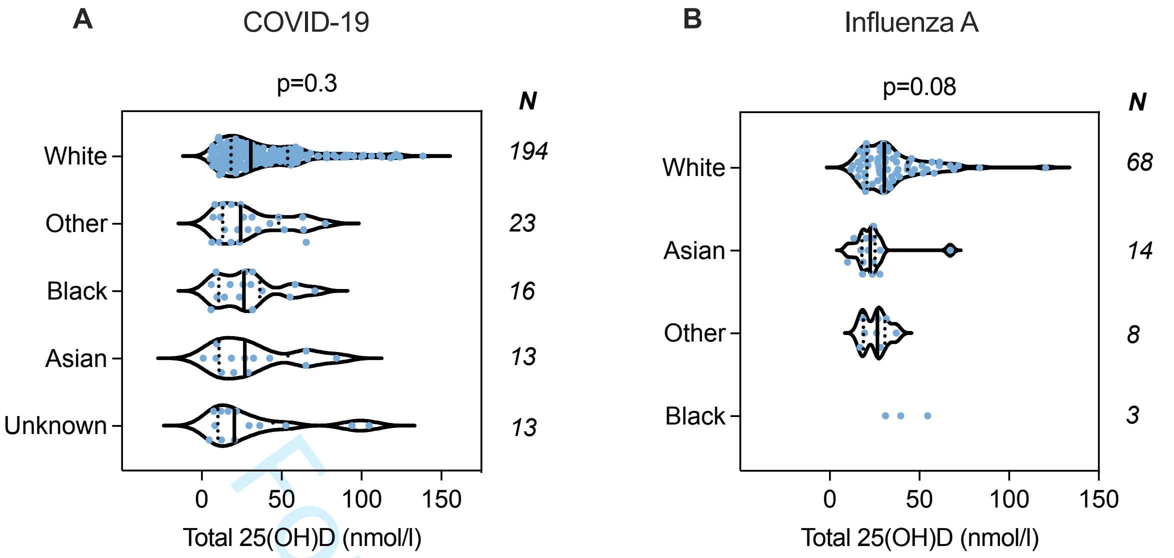

Supplementary Figure 1: Total 25(OH)D concentration stratified by ethnicity
(A) COVID-19 and **(B)** influenza A. The solid line within the violin plot represents the median and the dotted lines represent the interquartile range. Groups ≤5 are shown as individual data points. Groups were compared by ANOVA. N refers to the number of patients in each group.

For peer review only

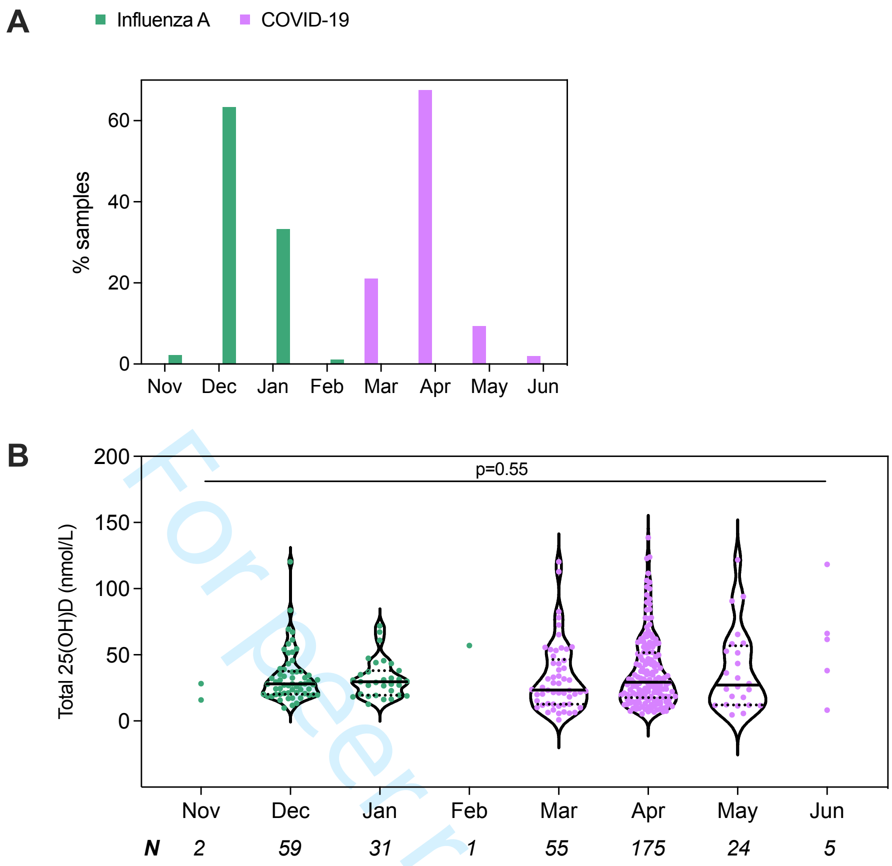

Supplementary Figure 2: Total 25(OH)D stratified by months of the year
(A) Month of the year during which samples were obtained from people with influenza A (2009-2011) and COVID-19 (2020). **(B)** Total 25(OH)D concentrations stratified by month of the year the sample was obtained. Groups compared by Kruskal-Wallis test. The solid line within the violin plot represents the median and the dotted lines represent the interquartile range. N refers to the number of samples for each month. Groups ≤5 are shown as individual data points.

For peer review only

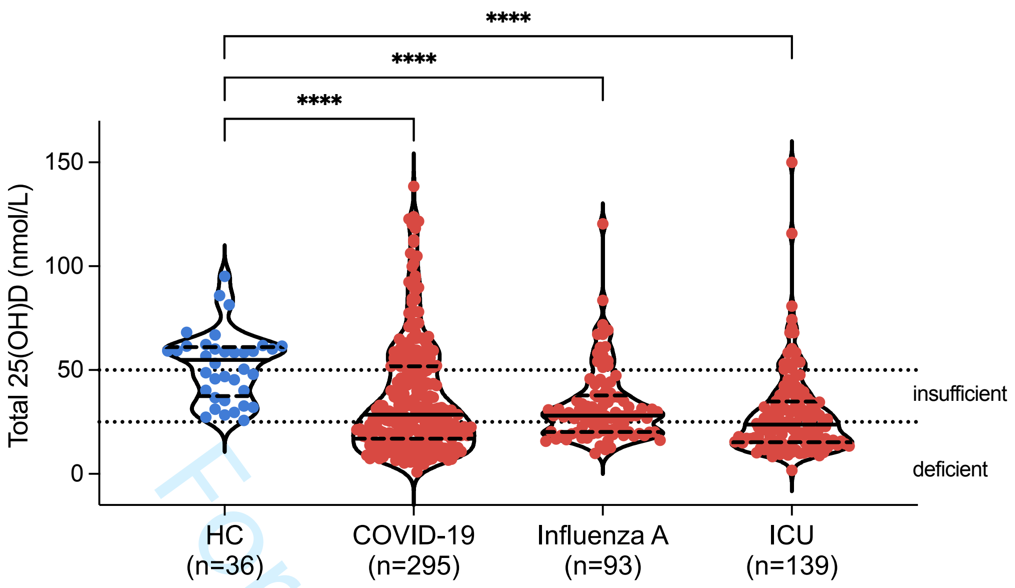

Supplementary Figure 3: Total 25(OH)D in patient cohorts compared to healthy controls
Total 25(OH)D measured in healthy controls ("HC", from the MOSAIC study recruited between June-September 2011), hospitalised patients with COVID-19 and influenza A, and non-selected critical illness survivors ("ICU"). The solid line within the violin plot represents the median and the dashed lines represent the interquartile range. Patient cohorts compared to healthy controls by Kruskal-Wallis test and Dunn's multiple comparisons test. **** p<0.0001

For peer review only

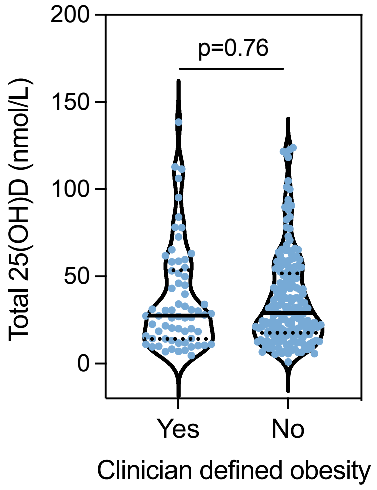

Supplementary Figure 4: Total 25(OH)D in patients with COVID-19 with/without obesity

Total 25(OH)D levels in hospitalised patients with COVID-19 with/without clinician defined obesity. Groups compared by Mann-Whitney test. The solid line within the violin plot represents the median and the dotted lines represent the interquartile range.

For peer review only

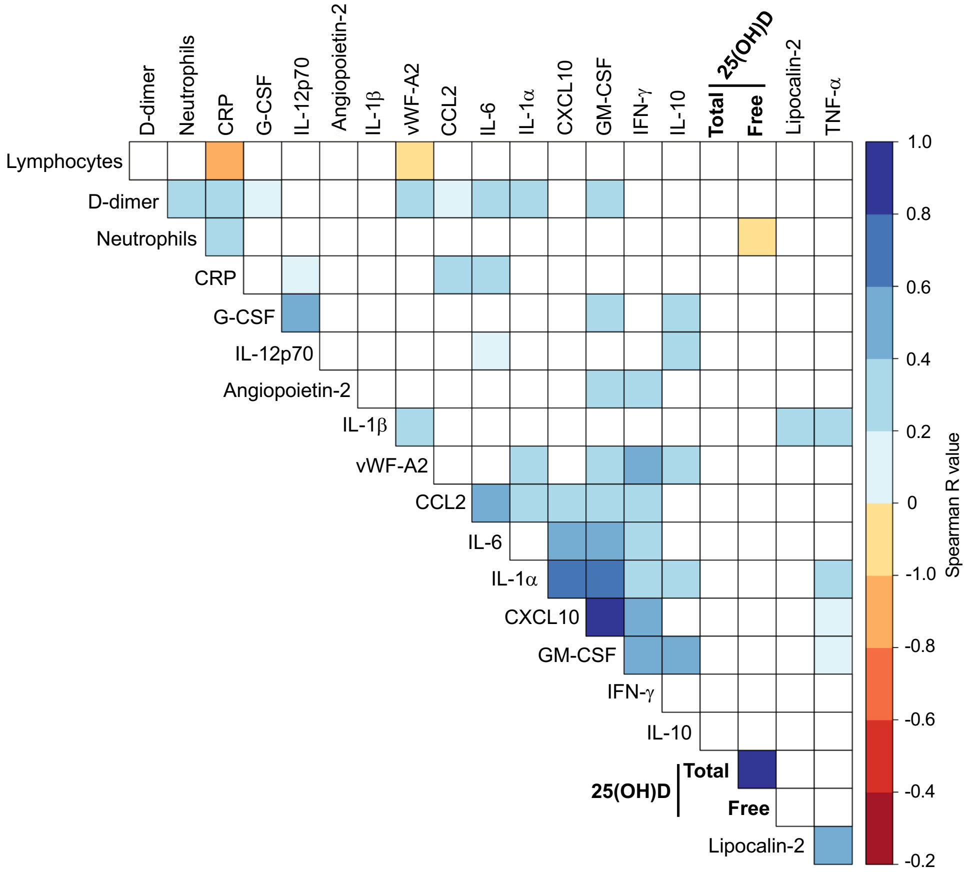

Supplementary Figure 5: Correlation analysis of 25(OH)D and inflammatory mediators
Correlogram of concentrations of plasma inflammatory mediators associated with COVID-19 severity and 25(OH)D (free and total). Cells with a correlation with p<0.05 (after correction for multiple comparisons) are shaded according to the Spearman R value. Inflammatory mediator measurements were available for 66 patients. Analysis was performed using the *corrplot* package in R.

For peer review only

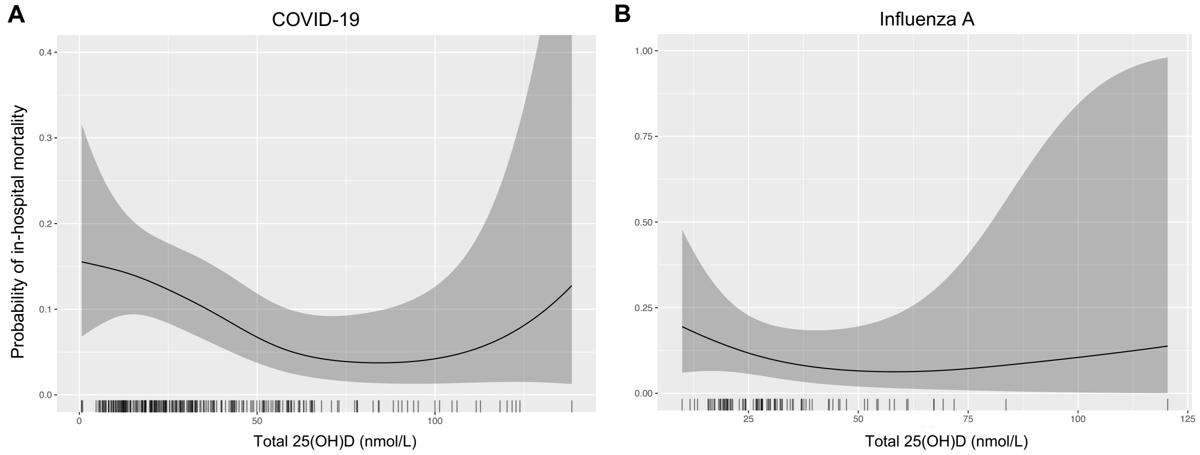

Supplementary Figure 6: Total 25(OH)D concentration and in-hospital mortality in COVID-19 and influenza A.

Smoothed predicted probability of in-hospital mortality vs. total 25(OH)D concentration (with other co-variates at mean values) from the binary logistic regression multivariable models for hospitalised people with **(A)** COVID-19 and **(B)** influenza A. Grey ribbon represents estimated 95% confidence interval and the x-axis ticks show observations.

SUPPLEMENTARY METHODS

Definition of vitamin D status

A total 25(OH)D concentration of 50nmol/L is the value widely used to define vitamin D sufficiency since experimental studies have shown that this is the concentration at which parathyroid hormone concentrations plateau [1,2]. Furthermore, based on evidence from the Institute of Medicine (IOM) and the Scientific Advisory Committee on Nutrition (SACN) which demonstrate an increased risk of poor muscoskeletal health with 25(OH)D levels between 20-30 nmol/L, the Royal Osteoporosis Society guidelines (which advice on testing and treatment of vitamin D in primary care in for the NHS), suggest that plasma 25(OH)D of 25-50 nmol/L may be inadequate in some people [3].

1. Sai AJ, Walters RW, Fang X, Gallagher JC. Relationship between Vitamin D, Parathyroid Hormone, and Bone Health. J Clin Endocrinol Metab **2011**; 96:E436–E446. Available at: https://doi.org/10.1210/jc.2010-1886.
2. Lips P. Vitamin D Deficiency and Secondary Hyperparathyroidism in the Elderly: Consequences for Bone Loss and Fractures and Therapeutic Implications. Endocr Rev **2001**; 22:477–501. Available at: https://doi.org/10.1210/edrv.22.4.0437.
3. Francis R, Aspray T, Fraser W, et al. Vitamin D and Bone Health : A Practical Clinical Guideline for Patient Management. 2018. Available at: https://strwebprdmedia.blob.core.windows.net/media/ef2ideu2/ros-vitamin-d-and-bone-health-in-adults-february-2020.pdf.

For peer review only

STROBE 2007 (v4) Statement—Checklist of items that should be included in reports of *cross-sectional studies*

Section/Topic	Item #	Recommendation	Reported on page #
**Title and abstract**	1	(*a*) Indicate the study's design with a commonly used term in the title or the abstract	1
		(*b*) Provide in the abstract an informative and balanced summary of what was done and what was found	3
**Introduction**			
Background/rationale	2	Explain the scientific background and rationale for the investigation being reported	5
Objectives	3	State specific objectives, including any prespecified hypotheses	5-6
**Methods**			
Study design	4	Present key elements of study design early in the paper	5
Setting	5	Describe the setting, locations, and relevant dates, including periods of recruitment, exposure, follow-up, and data collection	7-8
Participants	6	(*a*) Give the eligibility criteria, and the sources and methods of selection of participants	7-8
Variables	7	Clearly define all outcomes, exposures, predictors, potential confounders, and effect modifiers. Give diagnostic criteria, if applicable	9
Data sources/ measurement	8*	For each variable of interest, give sources of data and details of methods of assessment (measurement). Describe comparability of assessment methods if there is more than one group	8-9
Bias	9	Describe any efforts to address potential sources of bias	NA
Study size	10	Explain how the study size was arrived at	NA
Quantitative variables	11	Explain how quantitative variables were handled in the analyses. If applicable, describe which groupings were chosen and why	9
Statistical methods	12	(*a*) Describe all statistical methods, including those used to control for confounding	9
		(*b*) Describe any methods used to examine subgroups and interactions	NA
		(*c*) Explain how missing data were addressed	No missing data
		(*d*) If applicable, describe analytical methods taking account of sampling strategy	9-10
		(*e*) Describe any sensitivity analyses	NA
**Results**			

For peer review only

Participants	13*	(a) Report numbers of individuals at each stage of study—eg numbers potentially eligible, examined for eligibility, confirmed eligible, included in the study, completing follow-up, and analysed	11
		(b) Give reasons for non-participation at each stage	NA
		(c) Consider use of a flow diagram	NA
Descriptive data	14*	(a) Give characteristics of study participants (eg demographic, clinical, social) and information on exposures and potential confounders	11, Table 1
		(b) Indicate number of participants with missing data for each variable of interest	None
Outcome data	15*	Report numbers of outcome events or summary measures	11, Table 1
Main results	16	(*a*) Give unadjusted estimates and, if applicable, confounder-adjusted estimates and their precision (eg, 95% confidence interval). Make clear which confounders were adjusted for and why they were included	Yes, throughout
		(*b*) Report category boundaries when continuous variables were categorized	8-9
		(*c*) If relevant, consider translating estimates of relative risk into absolute risk for a meaningful time period	NA
Other analyses	17	Report other analyses done—eg analyses of subgroups and interactions, and sensitivity analyses	NA
**Discussion**			
Key results	18	Summarise key results with reference to study objectives	18
Limitations	19	Discuss limitations of the study, taking into account sources of potential bias or imprecision. Discuss both direction and magnitude of any potential bias	20
Interpretation	20	Give a cautious overall interpretation of results considering objectives, limitations, multiplicity of analyses, results from similar studies, and other relevant evidence	20
Generalisability	21	Discuss the generalisability (external validity) of the study results	20
**Other information**			
Funding	22	Give the source of funding and the role of the funders for the present study and, if applicable, for the original study on which the present article is based	21-22

*Give information separately for cases and controls in case-control studies and, if applicable, for exposed and unexposed groups in cohort and cross-sectional studies.

Note: An Explanation and Elaboration article discusses each checklist item and gives methodological background and published examples of transparent reporting. The STROBE checklist is best used in conjunction with this article (freely available on the Web sites of PLoS Medicine at http://www.plosmedicine.org/, Annals of Internal Medicine at http://www.annals.org/, and Epidemiology at http://www.epidem.com/). Information on the STROBE Initiative is available at www.strobe-statement.org.

For peer review only

Vitamin D insufficiency in COVID-19 and influenza A, and critical illness survivors: a cross-sectional study

Journal:	*BMJ Open*
Manuscript ID	bmjopen-2021-055435.R1
Article Type:	Original research
Date Submitted by the Author:	23-Aug-2021
Complete List of Authors:	Hurst, Emma A.; University of Edinburgh<br>Mellanby, Richard; The University of Edinburgh<br>Handel, Ian; University of Edinburgh<br>Griffith, David; Royal Infirmary of Edinburgh, Anaesthesia, Critical Care and Pain<br>Rossi, Adriano; University of Edinburgh MRC Centre for Inflammation Research, MRC Centre for Inflammation Research<br>Walsh, Timothy; The University of Edinburgh<br>Shankar-Hari, Manu; Guys and Guy's and St Thomas' Hospital NHS Foundation Trust, Intensive Care Unit; Kings College, London, Division of Asthma, Allergy and Lung Biology<br>Dunning, Jake; University of Oxford<br>Homer, Natalie Z.; University of Edinburgh<br>Denham, Scott G.; University of Edinburgh<br>Devine, Kerri; University of Edinburgh<br>Holloway, Paul A.; Imperial College London, Department of Metabolism, Digestion and Reproduction<br>Moore, Shona C.; University of Liverpool<br>Thwaites, Ryan S.; Imperial College London<br>Samanta, Romit J.; University of Cambridge, Department of Medicine<br>Summers, Charlotte; University of Cambridge, Department of Medicine<br>Hardwick, Hayley E.; University of Liverpool<br>Oosthuyzen, Wilna; University of Edinburgh<br>Turtle, Lance; University of Liverpool, NIHR Health Protection Research Unit in emerging and zoonotic infections, Institute of Infection, Veterinary and Ecological Sciences; Liverpool University Hospitals NHS Foundation Trust, Tropical and Infectious Disease Unit<br>Semple, Malcolm G; University of Liverpool, Institute of Translational Medicine<br>Openshaw, PJ; Imperial College London<br>Baille, J; Roslin Institute, Division of Genetics and Genomics<br>Russell, Clark; University of Edinburgh MRC Centre for Inflammation Research
**Primary Subject Heading**:	Infectious diseases
Secondary Subject Heading:	Immunology (including allergy), Intensive care, Respiratory medicine
Keywords:	COVID-19, INTENSIVE & CRITICAL CARE, Respiratory infections < THORACIC MEDICINE, IMMUNOLOGY

SCHOLARONE™
Manuscripts

I, the Submitting Author has the right to grant and does grant on behalf of all authors of the Work (as defined in the below author licence), an exclusive licence and/or a non-exclusive licence for contributions from authors who are: i) UK Crown employees; ii) where BMJ has agreed a CC-BY licence shall apply, and/or iii) in accordance with the terms applicable for US Federal Government officers or employees acting as part of their official duties; on a worldwide, perpetual, irrevocable, royalty-free basis to BMJ Publishing Group Ltd ("BMJ") its licensees and where the relevant Journal is co-owned by BMJ to the co-owners of the Journal, to publish the Work in this journal and any other BMJ products and to exploit all rights, as set out in our licence.

The Submitting Author accepts and understands that any supply made under these terms is made by BMJ to the Submitting Author unless you are acting as an employee on behalf of your employer or a postgraduate student of an affiliated institution which is paying any applicable article publishing charge ("APC") for Open Access articles. Where the Submitting Author wishes to make the Work available on an Open Access basis (and intends to pay the relevant APC), the terms of reuse of such Open Access shall be governed by a Creative Commons licence – details of these licences and which Creative Commons licence will apply to this Work are set out in our licence referred to above.

Other than as permitted in any relevant BMJ Author's Self Archiving Policies, I confirm this Work has not been accepted for publication elsewhere, is not being considered for publication elsewhere and does not duplicate material already published. I confirm all authors consent to publication of this Work and authorise the granting of this licence.

For peer review only

Vitamin D insufficiency in COVID-19 and influenza A, and critical illness survivors: a cross-sectional study

Emma A. Hurst[1,2], Richard J. Mellanby[1], Ian Handel[1], David M. Griffith[3], Adriano G. Rossi[4], Timothy S. Walsh[4], Manu Shankar-Hari[5], Jake Dunning[6], Natalie Z. Homer[2], Scott G. Denham[2], Kerri Devine[2], Paul A. Holloway[7], Shona C. Moore[8], Ryan S. Thwaites[9], Romit J. Samanta[10], Charlotte Summers[10], Hayley E. Hardwick[8], Wilna Oosthuyzen[11], Lance Turtle[7,12], Malcolm G. Semple[8,13], Peter J.M. Openshaw[9], J. Kenneth Baillie[11] and Clark D. Russell[4,11], on behalf of the ISARIC4C investigators

[1]The Roslin Institute and Royal (Dick) School of Veterinary Studies, University of Edinburgh, Edinburgh, U.K.

[2]Mass Spectrometry Core, Edinburgh Clinical Research Facility, Queen's Medical Research Institute, University of Edinburgh, Edinburgh, U.K.

[3]Molecular, Genetic and Population Health Sciences, University of Edinburgh, Edinburgh, U.K.

[4]University of Edinburgh Centre for Inflammation Research, The Queen's Medical Research Institute, Edinburgh, U.K.

[5]Peter Gorer Department of Immunobiology, School of Immunology & Microbial Sciences, Kings College London, U.K.

[6]Centre for Tropical Medicine and Global Health, University of Oxford, Oxford, U.K.

[7]Faculty of Medicine, Department of Metabolism, Digestion and Reproduction, Imperial College London, U.K.

[8]NIHR Health Protection Research Unit in Emerging and Zoonotic Infections, Institute of Infection, Veterinary and Ecological Sciences, Faculty of Health and Life Sciences, University of Liverpool, Liverpool, U.K.

[9]National Heart and Lung Institute, Imperial College London, U.K.

[10]Department of Medicine, University of Cambridge, U.K.

For peer review only

[11] Division of Genetics and Genomics, Roslin Institute, University of Edinburgh, Edinburgh, U.K.

[12]Tropical and infectious disease unit, Liverpool University Hospitals NHS Foundation Trust (member of Liverpool Health Partners), U.K.

[13]Respiratory Medicine, Alder Hey Children's Hospital, Liverpool, U.K.

Corresponding author:

Clark D Russell. University of Edinburgh Centre for Inflammation Research, The Queen's Medical Research Institute, 47 Little France Crescent, Edinburgh, U.K. EH16 4TJ. clark.russell@ed.ac.uk

Running title: Vitamin D in COVID-19, IAV & critical illness

Word counts:

Abstract: 266

Main: 3545

For peer review only

ABSTRACT

Objectives: The steroid hormone vitamin D has roles in immunomodulation and bone health. Insufficiency is associated with susceptibility to respiratory infections. We report 25(OH)D measurements in hospitalised people with COVID-19 and influenza A, and survivors of critical illness, to test the hypotheses that vitamin D insufficiency scales with illness severity and persists in survivors.

Design: Cross-sectional study

Setting and Participants: Plasma was obtained from 295 hospitalised people with COVID-19 (ISARIC/WHO CCP-UK study), 93 with influenza A (MOSAIC study, during the 2009-10 H1N1 pandemic), and 139 survivors of non-selected critical illness (prior to COVID-19 pandemic). Total 25(OH)D was measured by liquid chromatography-tandem mass spectrometry. Free 25(OH)D was measured by ELISA in COVID-19 samples.

Outcome measures: Receipt of invasive mechanical ventilation (IMV) and in-hospital mortality.

Results: Vitamin D insufficiency (total 25(OH)D 25-50 nmol/L) and deficiency (<25nmol/L) were prevalent in COVID-19 (29.3% and 44.4% respectively), influenza A (47.3% and 37.6%) and critical illness survivors (30.2% and 56.8%). In COVID-19 and influenza A, total 25(OH)D measured early in illness was lower in patients who received IMV (19.6 vs. 31.9 nmol/L, $p<0.0001$ and 22.9 vs. 31.1 nmol/L, $p=0.0009$ respectively). In COVID-19, biologically-active free 25(OH)D correlated with total 25(OH)D, was lower in patients who received IMV, but was not associated with selected circulating inflammatory mediators.

Conclusions: Vitamin D deficiency/insufficiency was present in the majority of hospitalised patients with COVID-19 or influenza A, correlated with severity and persisted in critical illness survivors at concentrations expected to disrupt bone metabolism. These findings support early supplementation trials to determine if insufficiency is causal in progression to severe disease, and investigation of longer-term bone health outcomes.

KEYWORDS: Vitamin D; Free 25-hydroxyvitamin-D; COVID-19; critical illness; influenza A.

For peer review only - http://bmjopen.bmj.com/site/about/guidelines.xhtml

STRENGTHS AND LIMITATIONS OF THIS STUDY

- Liquid chromatography-tandem mass spectrometry was used to quantify 25(OH)D in plasma samples from well characterised hospitalised people with COVID-19 and influenza A, and survivors of non-selected critical illness.
- Biologically active free 25(OH)D was measured by ELISA in COVID-19 plasma samples for the first time.
- Samples from people with COVID-19 and influenza A were obtained early in the course of disease.
- Binary logistic regression multivariable models were used to assess the association of plasma 25(OH)D concentration with outcomes in COVID-19 and influenza A, correcting for other known relevant covariates.
- The observational nature of the study means it is not known whether vitamin D status led to poor clinical outcome or was a consequence of illness severity.

For peer review only

INTRODUCTION

Vitamin D metabolites contribute to bone metabolism, calcium homeostasis and immunomodulation. Vitamin D is a steroid pre-pro-hormone which is converted to the main circulating form 25-hydroxy-vitamin D (25(OH)D), and subsequently to the active hormone 1,25 dihydroxy-vitamin D (1,25(OH)$_2$D). This second activation step occurs in the kidney, modulated by parathyroid hormone (PTH), for "endocrine" calciotropic effects, and also under local control within extra-renal tissues, including immune cells, for direct action. These "intracrine" actions on immune cells mediate anti-microbial and anti-inflammatory effects(1). The majority of 25(OH)D circulates bound to proteins, principally vitamin D binding protein (85-90%), and the relatively small unbound ("free") fraction is available to immune cells(2).

In the context of infectious diseases, vitamin D insufficiency (routinely determined by total 25(OH)D measurement) is associated with increased incidence and severity of respiratory tract infections(3-5) including coronavirus disease 2019 (COVID-19)(6, 7). A geographic association between vitamin D deficiency prevalence and COVID-19 incidence and mortality has been reported(8). Free 25(OH)D has not yet been investigated in COVID-19, but this is required to fully understand vitamin D status during acute illness and any associations with systemic inflammation(9). Clinical trials of vitamin D supplementation in respiratory diseases have returned mixed results(10-13). Potential beneficial effects of vitamin D supplementation may be pathogen-specific, and dependent upon timing and route of administration. In addition to an interest in modifying acute illness outcomes, longer term effects on bone health warrant consideration as critical illness is associated with loss of bone mineral density after recovery(14).

In this cross-sectional study, we report measurements of total and free 25(OH)D in hospitalised people with COVID-19, and total 25(OH)D in hospitalised people with influenza A and survivors of critical

For peer review only

illness. We use these three datasets to test the hypotheses that vitamin D insufficiency in severe respiratory virus infections scales with severity and persists in survivors of critical illness.

For peer review only

METHODS

Patients and sampling

COVID-19

The ISARIC WHO Clinical Characterization Protocol for Severe Emerging Infections in the UK (CCP-UK) is an ongoing prospective cohort study of hospitalized patients with COVID-19, which is recruiting in 308 hospitals in England, Scotland, Wales and Northern Ireland (National Institute for Health Research Clinical Research Network Central Portfolio Management System ID: 14152), delivered by the ISARIC Coronavirus Clinical Characterisation Consortium (ISARIC4C) investigators. The protocol, revision history, case report form and consent forms are available online at isaric4c/net. The ISARIC/WHO CCP-UK study was registered at https://www.isrctn.com/ISRCTN66726260 and designated an Urgent Public Health Research Study by the National Institute for Health Research UK. A prespecified case report form was used to collect data on patient characteristics, medical interventions received and outcomes, as previously reported(15).

Influenza A

Hospitalised patients with influenza A were recruited between 2009 and 2010 (the first and second H1N1 pandemic waves) and 2011 (the first post-pandemic season) by the MOSAIC (Mechanisms of Severe Acute Influenza Consortium) investigators.

Non-selected critical illness survivors

For peer review only

We include a post-hoc analysis of the RECOVER trial of intensive rehabilitation after critical illness(16). Full eligibility criteria have been published previously; briefly, adults were recruited who had received invasive mechanical ventilation (IMV) for at least 48 hours and were considered well enough for discharge from the intensive care unit (ICU). Patients gave additional consent for participation in a biomarker sub-study and blood samples were collected at ICU discharge(17).

Ethics Approvals

Ethical approval for the ISARIC/WHO CCP-UK study (COVID-19) was given by the South Central Oxford C Research Ethics Committee in England (13/SC/0149), the Scotland A Research Ethics Committee (20/SS/0028), and the WHO Ethics Review Committee (RPC571 and RPC572, 25 April 2013). Ethical approval for the MOSAIC study (influenza A) was given by the NHS National Research Ethics Service, Outer West London Research Ethics Committee (09/H0709/52, 09/MRE00/67), as previously reported(18). Participants gave informed consent.

Patient and Public Involvement

There was no patient or public involvement in this study.

LC-MS/MS methods for total 25(OH)D analysis

EDTA plasma concentrations (on samples obtained on the day of enrolment to the study) of 25(OH)D2 and 25(OH)D3 isoforms were measured by liquid chromatography tandem mass spectrometry (LC-MS/MS) and summed to derive the total 25(OH)D concentrations presented in the results. For patients with COVID-19 and critical illness survivors, analysis was performed by the Vitamin D Animal Laboratory (VitDAL) using an assay which has been certified as proficient by the international Vitamin

For peer review only - http://bmjopen.bmj.com/site/about/guidelines.xhtml

D Quality Assessment Scheme (DEQAS) and described in detail in an earlier manuscript, using 200μL plasma(19). Inter-assay precision (coefficient of variation) of this method was <11.5% for both 25(OH)D2 and 25(OH)D3 analytes (Supplementary Table 1). For patients with influenza A, analysis was performed using another LC-MS/MS method at a separate clinical biochemistry laboratory. Inter-assay precision of this method was <11% for 25(OH)D2 and <10% for 25(OH)D3 (Supplementary Table 1). Full LC-MS/MS methods are presented in Supplementary Table 1.

Definition of vitamin D status

In addition to the absolute total 25(OH)D concentration, the relationship between vitamin D status and outcomes is often explored using a total 25(OH)D cut off of 50 nmol/L to define populations that are vitamin D sufficient(20). In this study, total 25(OH)D >50nmol/L is reported as “sufficient”, 25–50 nmol/L as “insufficient” and <25 nmol/L as “deficient” (see Supplementary Methods).

Free 25(OH)D ELISA

Free 25(OH)D was measured using the Free 25OH Vitamin D ELISA (DIAsource ImmunoAssays® S.A, Belgium), following manufacturer’s instructions, using 10μL of plasma. Absorbance was measured at 450nm against a reference filter set at 630nm using the Tecan Sunrise™ Microplate Reader (TECAN). GraphPad Prism (version 7.0e for Mac OS X) was used to perform a 4-parameter logistic function to create the calibration curve in order to read the mean concentration of duplicate samples. The lower limit of detection (LLOD) of the assay was 2.4pg/mL. The intra-assay repeatability (coefficient of variation (CV) was ≤5.5% across 3 concentrations (low, mid and high concentrations on the standard curve) and the inter-assay precision (CV) was <6.5% across the 3 concentrations, calculated based on CLSI EP05-A3 and reported in the manufacturer’s guidelines. Two control samples (a low and high concentration) were analysed in each batch in duplicate and data were only reported for the batch if

For peer review only

For peer review only - http://bmjopen.bmj.com/site/about/guidelines.xhtml

the results of the controls were within the acceptance range outlined on each control sample vial. Each calibrator, control and patient sample were assessed in duplicate and results only reported if the CV of the replicates was <10%.

Statistical analysis

For univariable analyses, the Shapiro-Wilk test was used to test for normal data distribution then appropriate tests, specified in the text, were used for comparisons. Associations between covariates and outcomes in COVID-19 and influenza A were assessed with binary logistic regression multivariable models. Sex, age, illness duration at time of sampling and comorbidity count were chosen as covariates. The comorbidity count was derived from the same comorbidities (Table 1) from the two cohorts. To allow for potential non-linear relationship between predictors and the probability of an outcome, the models included smoothed thin plate regression spline terms for age, illness duration at time of sampling, comorbidity count and 25(OH)D concentrations. Multivariable models were estimated using the *gam()* function of the R *mgcv* package using the default, thin plate regression smoothers(21, 22). The upper limit of smoother dimensionality was set to 9 for all variables excluding the comorbidity count where it was set to 7 as this variable was discrete with 7 levels. Smoother parameters were estimated with restricted maximum likelihood. 25(OH)D concentrations were below the LLOD for 92 patients (free) and 2 patients (total) in the COVID-19 cohort. For the regression models, 25(OH)D values for these patients were imputed as the LLOD for the relevant analyte divided by the square root of two(23). As this is a commonly used but arbitrary method the regression analysis was repeated using zero and the LLOD as imputed values to assess sensitivity of the result to this assumption. Effects for categorical covariates are reported as odds ratios; smoothed continuous covariates are reported graphically. Statistical analyses were conducted in R using the *mgcv*, *tidyverse* and *gratia* packages.

For peer review only - http://bmjopen.bmj.com/site/about/guidelines.xhtml

RESULTS

Patient characteristics

Samples were obtained from 259 people hospitalised due to COVID-19 and 93 people hospitalised due to influenza A. Samples were also obtained from 139 critical illness survivors (prior to the COVID-19 pandemic) at time of ICU discharge. Patient characteristics, including sampling time after symptom onset, are shown in Table 1. For COVID-19 patients, samples were obtained a median of 3 days (IQR 2-6) after hospital admission. Patients with influenza A were younger, more likely to be female and more likely to have asthma compared to the other cohorts. Receipt of IMV and in-hospital mortality did not differ between COVID-19 and influenza A. The WHO ordinal severity scale scores for people with COVID-19 are shown in Supplementary Figure 1, illustrating that the cohort is representative of the full spectrum of disease severity in hospitalised people. Details on ethnicity were available for the COVID-19 and influenza A cohorts. No differences in total 25(OH)D were observed between ethnic groups, but only small numbers of participants were from non-white groups (COVID-19 65/259, influenza A 25/93; Supplementary Figure 2). All samples from people with influenza A were collected between the months of November to February (63.4% in December), whereas all samples from people with COVID-19 were collected between March to June (67.6% in April). However, the distribution of total 25(OH)D measurements did not differ when stratified by month (Supplementary Figure 3). Total 25(OH)D concentration was lower in all three patient cohorts when compared to healthy controls (n=36; Supplementary Figure 4), but the healthy control samples were obtained between the months of June to September.

For peer review only

For peer review only - http://bmjopen.bmj.com/site/about/guidelines.xhtml

Table 1: Characteristics of included patients

Characteristic	COVID-19 (n=259)	Influenza A (n=93)	Critical illness survivors (n=139)	p-value[a]
**Demographics**				
Age at admission, years[b]	63 (52-73)	43 (29-50)	63 (53-70)	<0.0001
Male sex	175 (67.6)	47 (50.5)	85 (61.2)	0.01
Day of illness at time of sampling[b]	10 (6-16)	7 (4-11)	11 (6-18)[c]	<0.001[d]
**Co-morbidities**				
Diabetes mellitus	66 (25.5)	10 (10.8)	23 (16.5)	0.005
Chronic cardiac disease	57 (22.4)	17 (18.3)	15 (10.8)	0.02
Obesity, clinician defined	44 (18.7)	23 (24.7)	28 (20.1)	0.3
Asthma	41 (16.1)	33 (35.5)	26 (18.7)	0.0002
Chronic lung disease, not asthma	35 (13.8)	12 (12.9)	24 (17.3)	0.5
Chronic kidney disease	25 (9.9)	4 (4.3)	NA	0.1
Neoplasia	14 (5.6)	9 (9.7)	NA	0.2
Moderate or severe liver disease	3 (1.2)	4 (4.3)	NA	0.08
**Illness severity**				
Admission to critical care	106 (40.9)	32 (34.4)	139 (100)	0.3[d]
Invasive mechanical ventilation	67 (25.9)	29 (31.2)	139 (100)	0.3[d]
In-hospital mortality	52 (20.1)	12 (12.9)	4 (2.9)[e]	0.2[d]
**Total plasma 25(OH)D**				
Median (IQR) nmol/L	28.5 (17.1-51.9)	28.1 (20.2-37.9)	23.7 (15.3-34.9)	0.01
Status				
Sufficient (>50 nmol/L)	68 (26.3)	14 (15.1)	18 (12.9)	
Insufficient (25-50 nmol/L)	76 (29.3)	44 (47.3)	42 (30.2)	0.0002
Deficient (<25 nmol/L)	115 (44.4)	35 (37.6)	79 (56.8)	

Data are number (%) unless otherwise stated.

[a]Kruskal-Wallis, Mann-Whitney or Chi^2 test as appropriate

[b]median (interquartile range)

[c]length of ICU stay

[d]comparing COVID-19 and influenza A

For peer review only - http://bmjopen.bmj.com/site/about/guidelines.xhtml
For peer review only

[e] death after discharge from ICU

For peer review only

For peer review only - http://bmjopen.bmj.com/site/about/guidelines.xhtml

Total 25(OH)D correlates with severity in COVID-19

The majority of COVID-19 patients had total 25(OH)D concentrations indicative of vitamin D insufficiency (29.3%) or deficiency (44.4%; Table 1). Total 25(OH)D was lower in men than women (median 26.8 [IQR 14.1-47.4] vs. 31.7 [20.1-63.8] nmol/L, p=0.01) and weakly positively correlated with increased age (Pearson *r* 0.25, p<0.0001).

When stratified by receipt of IMV as a marker of illness severity, total 25(OH)D differed significantly with a median concentration of 19.6 nmol/L (IQR 12.6-32.3) in patients receiving IMV compared to 31.9 nmol/L (IQR 20.0-58.3) in the remainder of the cohort (p<0.0001, Figure 1A). When total 25(OH)D was stratified by associated vitamin D status, patients receiving IMV were more likely to be insufficient/deficient (Figure 1A). Amongst patients who received IMV, 64.2% (43/67) were deficient and 26.9% (18/67) were insufficient. Total 25(OH)D concentration was also associated with in-hospital mortality (median 23.2 nmol/L [IQR 15.4-39.9] in non-survivors vs. 29.5 nmol/L [IQR 17.2-55.4] in survivors, p=0.01). Total 25(OH)D concentrations were divided into quartiles and the proportion of patients who received IMV was compared (Figure 1B). The lowest quartile (≤17.3nmol/L) had the highest proportion of patients receiving IMV (43.1%). The middle quartiles were similar (26.6 and 25.0%), with the highest 25(OH)D quartile (>51.8nmol/L) containing the lowest proportion receiving IMV (7.8%, Chi^2 p=0.0001).

Obesity is a risk factor for severity and mortality in COVID-19, and can be associated with vitamin D deficiency(15). However, there was no difference in total 25(OH)D concentration between patients with/without clinician defined obesity (Supplementary Figure 5). Inflammatory mediator measurements had previously been performed on plasma samples from 66 patients included in this study(24). Correlation matrix analysis demonstrated that total 25(OH)D was not significantly

For peer review only - http://bmjopen.bmj.com/site/about/guidelines.xhtml

associated with circulating markers of systemic inflammation demonstrated to be involved in COVID-19 pathogenesis (Supplementary Figure 6).

Multivariable analyses confirmed that total 25(OH)D concentration and vitamin D status (not sufficient) were both independently and negatively associated with receipt of IMV (Table 2, Figure 2A, Supplementary Table 2). Two patients had total 25(OH)D concentrations below the LOD; using zero and LOD, instead of LOD divided by the square root of two, had no substantive effect on significance of covariates or their effect sizes. Vitamin D status was also independently associated with in-hospital mortality, but total 25(OH)D concentration was not (Supplementary Table 2; Supplementary Figure 7A).

For peer review only

Table 2: Multivariable analyses of 25(OH)D concentration and outcomes

Variable	Odds ratio	p-value
**Total 25(OH)D**		
COVID-19: receipt of IMV		
Male sex	2.33 (1.13-4.78)	0.022
Comorbidity count[a]	-	0.487
**Total 25(OH)D**[a]	-	0.001
Day of illness[a]	-	0.386
Age[a]	-	0.061
Influenza A: receipt of IMV		
Male sex	2.22 (0.54 – 9.06)	0.27
Comorbidity count[a]	-	0.15
**Total 25(OH)D**[a]	-	0.016
Day of illness[a]	-	0.001
Age[a]	-	0.19
**Free 25(OH)D**		
COVID-19: receipt of IMV		
Male sex	2.53 (1.24-5.314)	0.011
Comorbidity count[a]	-	0.605
**Free 25(OH)D**[a]	-	0.006
Day of illness[a]	-	0.577
Age[a]	-	0.053
COVID-19: in-hospital mortality		
Male sex	2.78 (1.25-6.17)	0.012
Comorbidity count[a]	-	0.022
**Free 25(OH)D**[a]	-	0.025
Day of illness[a]	-	0.795
Age[a]	-	0.041

IMV: invasive mechanical ventilation

[a]smoothed

For peer review only - http://bmjopen.bmj.com/site/about/guidelines.xhtml

For peer review only

Total 25(OH)D correlates with severity in influenza A

We then extended these observations to total 25(OH)D concentrations measured in people hospitalised with influenza A. Total 25(OH)D was not associated with age (p=0.1) or sex (p=0.8). Similar to our findings in COVID-19, the majority of patients had total 25(OH)D concentrations indicative of vitamin D insufficiency (47.3%) or deficiency (37.6%; Table 1). When stratified by receipt of IMV, total 25(OH)D was lower in patients receiving IMV (median 22.9 nmol/L, IQR 18.0-29.8) compared to the remainder of the cohort (median 31.1 nmol/L, IQR 23.8-45.2, p=0.0009) and these patients were more likely to be vitamin D insufficient/deficient (Figure 1C). Total 25(OH)D was lower in non-survivors compared to survivors (median 22.1 nmol/L [IQR17.6-34.1] vs. 29.2 nmol/L [IQR 20.6-38.5]) but this was not statistically significant (p=0.2). Multivariable analysis confirmed an independent negative association between total 25(OH)D and receipt of IMV but not in-hospital mortality (Figure 2B, Table 2; Supplementary Table 3; Supplementary Figure 7B).

Vitamin D deficiency persists in survivors of critical illness

In survivors of non-selected critical illness, at the time of ICU discharge the median total 25(OH)D concentration was 22.9 nmol/L (IQR 14.6-34.6), similar to concentrations in patients with COVID-19/influenza A who required IMV or did not survive. The majority of patients had total 25(OH)D concentrations indicative of vitamin D deficiency (56.8%) or insufficiency (30.2%; Figure 1D and Table 1). Total 25(OH)D concentration was not associated with age (p=0.7), sex (p=0.7) or length of ICU stay (p=0.8). Measurements were not available from earlier in these patients' illnesses.

Free 25(OH)D correlates with severity in COVID-19

For peer review only - http://bmjopen.bmj.com/site/about/guidelines.xhtml

In patients with COVID-19, we found a strong correlation between free and total 25(OH)D concentrations (*r*=0.79, p<0.0001) (Figure 3A). Free 25(OH)D was lower in patients receiving IMV (median 2.4 pg/mL [IQR 2.4-3.4] vs. 3.6 pg/mL [IQR 2.4-5.7], p<0.0001; Figure 3B) but was not statistically different between survivors and non-survivors on univariable analysis (median 2.8 pg/mL [IQR 2.4-4.4] vs. 3.3 pg/mL [IQR 2.4-5.3], p=0.2). In multivariable analysis, free 25(OH)D was negatively associated with both receipt of IMV and in-hospital mortality (Figure 2B-D, Table 2). Free 25(OH)D was not associated with plasma inflammatory mediator concentrations (Supplementary Figure 6).

For peer review only

DISCUSSION

Vitamin D insufficiency was prevalent and scaled with severity in patients with COVID-19 and influenza A, and insufficiency persisted in survivors of critical illness. 73% of COVID-19 patients, 84% of influenza A patients and 87% of critical illness survivors were vitamin D insufficient/deficient, determined by total 25(OH)D measurement. We demonstrate evidence of a strong association between vitamin D status (insufficiency/deficiency) during illness and both COVID-19 severity (receipt of IMV) and in-hospital mortality, with relevant confounders such as sex, age, comorbidities and day of illness adjusted for. This observation was replicated in influenza A but the smaller sample size (n=93 compared to 259) limited multivariable analyses. For the first time, we demonstrate a similar strong negative association between free 25(OH)D and COVID-19 disease severity and mortality. The results from this study extend earlier findings from other observational studies reporting associations between vitamin D status and severe acute respiratory syndrome coronavirus 2 (SARS-CoV-2) infection and COVID-19 outcome(6, 7, 25-27).

Vitamin D may beneficially modulate the host response against SARS-CoV-2 via intracrine immune signalling. Vitamin D enhances intracellular pathogen clearance, primarily via the induction of autophagy(28). Importantly, the ability of macrophages to produce cathelicidin, which has anti-viral activity against influenza virus and respiratory syncytial virus, correlates with circulating 25(OH)D concentrations(29). Although anti-viral effects of vitamin D have not yet been demonstrated *in vitro* for SARS-CoV-2, they have been demonstrated for other bacterial and viral pathogens(30, 31). Consistent with vitamin D having a role in local immunomodulation, neither free nor total 25(OH)D correlated with circulating markers of systemic inflammation involved in COVID-19 pathogenesis (including CRP, IL-6 and GM-CSF(24)).

For peer review only - http://bmjopen.bmj.com/site/about/guidelines.xhtml

Evidence for the importance of free versus total 25(OH)D in relation to the mechanisms by which vitamin D exerts antimicrobial and anti-inflammatory functions have been demonstrated(32, 33). We now demonstrate that free 25(OH)D was negatively associated with COVID-19 severity and in-hospital mortality. Studies directly measuring free 25(OH)D and immune responses to infection or during critical illness are limited. In a study of 30 critically ill patients, supplementation with high dose vitamin D increased free 25(OH)D and plasma cathelicidin concentrations(34). Another study of 30 patients with sepsis reported similar results when they examined the effects of vitamin D supplementation on bioavailable (combined albumin-bound and free fraction) 25(OH)D and cathelicidin concentrations(35). Together, these findings suggest that low concentrations of free 25(OH)D may reduce the vitamin D-induced antimicrobial and anti-inflammatory response, compromising immune defences.

We found that 30.2% of patients surviving critical illness and requiring IMV (prior to the COVID-19 pandemic) were vitamin D insufficient and 56.8% were deficient. Vitamin D deficiency is common in critical illness with a reported prevalence of between 40-70% in observational studies of both adults and children worldwide(36, 37). Although some patients may enter ICU in a deficient state due to pre-existing disease and malnutrition, vitamin D metabolism is dysregulated in critical illness(38) and concentrations fall rapidly after ICU admission(39). The mechanistic link between acute illness and vitamin D deficiency is likely to be multi-factorial, including reduced dietary intake/absorption, reduced cutaneous synthesis due to lack of sunlight and wastage due to reductions in vitamin D binding protein(40). Furthermore, vitamin D insufficiency/deficiency has been associated with a range of poor outcomes in critical illness(37, 41-43). Vitamin D insufficiency leads to secondary hyperparathyroidism and a concentration of 50 nmol/L total 25(OH)D is required for optimum PTH concentrations(44). 87% of the critical illness survivors had total 25(OH)D <50 nmol/L, which would be associated with secondary hyperparathyroidism and the potential for associated loss of bone mineral density. Critical illness survivors suffer accelerated loss of bone mineral density in the year

For peer review only - http://bmjopen.bmj.com/site/about/guidelines.xhtml

after ICU discharge (compared to matched controls) and increased 10-year fracture risk(14). Our findings implicate vitamin D insufficiency in this process.

There is evidence that vitamin D supplementation can improve circulating total 25(OH)D concentrations in critically ill patients(34, 35, 45), but evidence of a beneficial effect on outcomes is less clear. High-dose vitamin D supplementation in COVID-19(13) and critical illness(45) has been shown to increase plasma 25(OH)D concentrations 7-days post-supplementation but no significant reduction in the length of hospital stay or acute outcomes including in-hospital mortality, admission to ICU or requirement for IMV were demonstrated(13, 45, 46). Longer term outcomes such as bone health have not been evaluated. Conversely, two reports of randomized trials of high-dose 25(OH)D3 (instead of vitamin D3 as in the above mentioned studies) on admission and then subsequent doses on either days 3, 7, then weekly (n=76)(47) or days 3, 7, 15, and 30(n=838)(48), were less likely to require ICU admission. We identified that vitamin D insufficiency was present early in the course of COVID-19 and influenza A (10 and 7 days after symptom onset respectively) indicating that timing of supplementation may be an important factor when designing future supplementation studies. We propose that future studies examining effects on disease progression should investigate the effects of vitamin D supplementation given earlier in the course of disease, closer to symptom onset rather than after hospitalisation. Evidence from an observational study of vitamin D supplementation usage supports this approach(49). In a cohort of 8297 people with SARS-CoV-2 test results available, habitual vitamin D supplement intake prior to the pandemic was associated with a reduced risk of a positive test result after correction for known confounders including demographics and co-morbidities. Furthermore, despite a decline in vitamin D following cardiothoracic surgery, post-operative outcomes (including organ dysfunction and mortality) are still associated with pre-operative vitamin D status(50). This suggests that supplementation prior to illness onset can still be expected to improve outcomes despite the fall in vitamin D concentration during acute illness. The longer-term effects of persistent vitamin D insufficiency/deficiency in survivors of critical illness also requires further

For peer review only - http://bmjopen.bmj.com/site/about/guidelines.xhtml

investigation especially in the context of bone health which could be independently evaluated using sequential measurement of bone turnover markers and serum PTH.

The current study has several important limitations. The observational design prevents any conclusions about a causal role for vitamin D status in poor clinical outcome being drawn. We cannot exclude the alternative explanation that the differences in vitamin D status were a consequence of illness severity. Although blood samples were obtained from people with COVID-19 and influenza A as soon after hospital admission as feasible, even these early measurements will still be subject to acute illness-related changes in vitamin D homeostasis. Healthy control data is presented (Supplementary Figure 4) but these samples were obtained between the months of June-September whereas samples from people with COVID-19 and influenza A were obtained between November-June (though no inter-month variation was observed). Data on pre- or in-hospital vitamin D supplementation was not available. Finally, longer term follow-up samples to assess vitamin D status in survivors of COVID-19 will be informative.

In conclusion, vitamin D deficiency/insufficiency was present in the majority of hospitalised patients with COVID-19 or influenza A and scaled with severity, highlighting that reduced concentrations of vitamin D is common to these disease states and distinct patient cohorts. For the first time, free and total 25(OH)D were studied in COVID-19 demonstrating consistent results. It is not clear whether vitamin D status led to poor clinical outcome or was a consequence of illness severity. Randomised trials will be necessary to determine whether a causal relationship exists between vitamin D early in the course of disease and development of critical illness. Since vitamin D deficiency/insufficiency persisted at concentrations expected to disrupt bone metabolism in critical illness survivors, investigation of longer-term bone health outcomes is also warranted.

For peer review only - http://bmjopen.bmj.com/site/about/guidelines.xhtml

ACKNOWLEDGEMENTS

The ISARIC4C work uses data provided by patients and collected by the NHS as part of their care and support. We are extremely grateful to the 2648 front-line NHS clinical and research staff and volunteer medical students who collected this data in challenging circumstances; and the generosity of the participants and their families for their individual contributions in these difficult times. Thanks to Joanna Simpson and Patricia Lee who were involved in sample management and free 25(OH)D data collection during the analytical process. Thanks to Catherine Duff and Sharon Hannah who assisted in the health and safety aspects of the project, and to the Clinical Biochemistry Department at Imperial College Healthcare NHS Trust for analysing the samples from influenza patients in 2011. We also acknowledge the support of Jeremy J Farrar (Wellcome Trust) and Nahoko Shindo (WHO).

COMPETING INTERESTS STATEMENT

RJM and EH are part of the VitDAL which provides a 25(OH)D assay service on a not-for-profit basis.

FUNDING

This work was supported by grants from: the National Institute for Health Research (NIHR) [award CO-CIN-01], the Medical Research Council [grant MC_PC_19059] and by the NIHR Health Protection Research Unit (HPRU) in Emerging and Zoonotic Infections at University of Liverpool in partnership with Public Health England (PHE), in collaboration with Liverpool School of Tropical Medicine and the University of Oxford [award 200907], NIHR HPRU in Respiratory Infections at Imperial College London with PHE [award 200927], Wellcome Trust and Department for International Development [215091/Z/18/Z], and the Bill and Melinda Gates Foundation [OPP1209135], and Liverpool Experimental Cancer Medicine Centre (Grant Reference: C18616/A25153), NIHR Biomedical Research Centre at Imperial College London [IS-BRC-1215-20013], EU Platform for European Preparedness Against (Re-) emerging Epidemics (PREPARE) [FP7 project 602525] and NIHR Clinical Research Network for providing infrastructure support for this research. LT is supported by a Wellcome Trust fellowship

For peer review only - http://bmjopen.bmj.com/site/about/guidelines.xhtml

[205228/Z/16/Z]. The MOSAIC study was supported by the Wellcome Trust (087805/Z/08/Z) and the Medical Research Council HIC-Vac network (MR/R005982/1). PJMO is supported by a NIHR Senior Investigator Award [award 201385]. For the purpose of Open Access, the authors have applied a CC BY public copyright licence to any Author Accepted Manuscript version arising from this submission. RJS is funded by a EMINENT GSK-NIHR research training fellowship supported by the Cambridge NIHR Biomedical Research Centre. The RECOVER trial was funded by the Chief Scientists Office Scotland (CZH/4/53), trial registration ISRCTN09412438. Professor Shankar-Hari is funded by the National Institute for Health Research Clinician Scientist Award (CS-2016-16-011). We acknowledge the financial support of NHS Research Scotland (NRS), for the Mass Spectrometry Core, Edinburgh Clinical Research Facility.

The views expressed in this publication are those of the author(s) and not necessarily those of the NHS, the National Institute for Health Research or the Department of Health and Social Care, or other funders.

DATA SHARING

Access to all data and samples collected by ISARIC4C are controlled by an Independent Data and Materials Access Committee composed of representatives of research funders, academia, clinical medicine, public health, and industry. The application process for access to the data is available on the ISARIC4C website (https://isaric4c.net).

For peer review only

AUTHOR CONTRIBUTIONS

Conceptualisation: EA Hurst, RJ Mellanby, CD Russell, L Turtle, PJM Openshaw, JK Baillie and MG Semple

Data curation: EA Hurst, DM Griffith, J Dunning and CD Russell

Formal analysis: EA Hurst, I Handel and CD Russell

Funding acquisition: RJ Mellanby, PJM Openshaw, JK Baillie and MG Semple

Investigation: EA Hurst, RJ Mellanby, I Handel, DM Griffith, AG Rossi, TS Walsh, M Shankar-Hari, NZ Homer, SG Denham, K Devine, PA Holloway, RS Thwaites, RJ Samanta, J Dunning, C Summers, L Turtle, PJM Openshaw, JK Baillie, MG Semple and CD Russell

Project administration: SC Moore, HE Hardwick and W Oosthuyzen

Data visualisation: I Handel and CD Russell

Supervision: RJ Mellanby, MG Semple, PJM Openshaw, JK Baillie and CD Russell

Writing original draft: EA Hurst and CD Russell

Writing review & editing: EA Hurst, RJ Mellanby, I Handel, DM Griffith, AG Rossi, TS Walsh, M Shankar-Hari, NZ Homer, SG Denham, K Devine, PA Holloway, RS Thwaites, RJ Samanta, J Dunning, C Summers, L Turtle, PJM Openshaw, JK Baillie, MG Semple and CD Russell

Validation: EA Hurst, RJ Mellanby and CD Russell

For peer review only

AUTHORSHIP SUPPLEMENT

ISARIC4C Investigators

Consortium Lead Investigator: J Kenneth Baillie. Chief Investigator: Malcolm G Semple. Co-Lead Investigator: Peter JM Openshaw. ISARIC Clinical Coordinator: Gail Carson. Co-Investigator: Beatrice Alex, Benjamin Bach, Wendy S Barclay, Debby Bogaert, Meera Chand, Graham S Cooke, Annemarie B Docherty, Jake Dunning, Ana da Silva Filipe, Tom Fletcher, Christopher A Green, Ewen M Harrison, Julian A Hiscox, Antonia Ying Wai Ho, Peter W Horby, Samreen Ijaz, Saye Khoo, Paul Klenerman, Andrew Law, Wei Shen Lim, Alexander J Mentzer, Laura Merson, Alison M Meynert, Mahdad Noursadeghi, Shona C Moore, Massimo Palmarini, William A Paxton, Georgios Pollakis, Nicholas Price, Andrew Rambaut, David L Robertson, Clark D Russell, Vanessa Sancho-Shimizu, Janet T Scott, Thushan de Silva, Louise Sigfrid, Tom Solomon, Shiranee Sriskandan, David Stuart, Charlotte Summers, Richard S Tedder, Emma C Thomson, AA Roger Thompson, Ryan S Thwaites, Lance CW Turtle, Rishi K Gupta, Maria Zambon. Project Manager: Hayley Hardwick, Chloe Donohue, Ruth Lyons, Fiona Griffiths, Wilna Oosthuyzen. Data Analyst: Lisa Norman, Riinu Pius, Thomas M Drake, Cameron J Fairfield, Stephen R Knight, Kenneth A Mclean, Derek Murphy, Catherine A Shaw. Data and Information System Manager: Jo Dalton, Michelle Girvan, Egle Saviciute, Stephanie Roberts, Janet Harrison, Laura Marsh, Marie Connor, Sophie Halpin, Clare Jackson, Carrol Gamble. Data Integration and Presentation: Gary Leeming, Andrew Law, Murray Wham, Sara Clohisey, Ross Hendry, James Scott-Brown. Material Management: William Greenhalf, Victoria Shaw, Sara McDonald. Patient Engagement: Seán Keating. Outbreak Laboratory Staff and Volunteers: Katie A. Ahmed, Jane A Armstrong, Milton Ashworth, Innocent G Asiimwe, Siddharth Bakshi, Samantha L Barlow, Laura Booth, Benjamin Brennan, Katie Bullock, Benjamin WA Catterall, Jordan J Clark, Emily A Clarke, Sarah Cole, Louise Cooper, Helen Cox, Christopher Davis, Oslem Dincarslan, Chris Dunn, Philip Dyer, Angela Elliott, Anthony Evans, Lorna Finch, Lewis WS Fisher, Terry Foster, Isabel Garcia-Dorival, William Greenhalf, Philip Gunning, Catherine Hartley, Rebecca L Jensen, Christopher B Jones, Trevor R Jones, Shadia Khandaker, Katharine King, Robyn T. Kiy, Chrysa Koukorava, Annette Lake, Suzannah Lant, Diane Latawiec, Lara

For peer review only

Lavelle-Langham, Daniella Lefteri, Lauren Lett, Lucia A Livoti, Maria Mancini, Sarah McDonald, Laurence McEvoy, John McLauchlan, Soeren Metelmann, Nahida S Miah, Joanna Middleton, Joyce Mitchell, Shona C Moore, Ellen G Murphy, Rebekah Penrice-Randal, Jack Pilgrim, Tessa Prince, Will Reynolds, P. Matthew Ridley, Debby Sales, Victoria E Shaw, Rebecca K Shears, Benjamin Small, Krishanthi S Subramaniam, Agnieska Szemiel, Aislynn Taggart, Jolanta Tanianis-Hughes, Jordan Thomas, Erwan Trochu, Libby van Tonder, Eve Wilcock, J. Eunice Zhang, Lisa Flaherty, Nicole Maziere, Emily Cass, Alejandra Doce Carracedo, Nicola Carlucci, Anthony Holmes, Hannah Massey. Edinburgh Laboratory Staff and Volunteers: Lee Murphy, Nicola Wrobel, Sarah McCafferty, Kirstie Morrice, Alan MacLean. Local Principal Investigators: Kayode Adeniji, Daniel Agranoff, Ken Agwuh, Dhiraj Ail, Erin L. Aldera, Ana Alegria, Brian Angus, Abdul Ashish, Dougal Atkinson, Shahedal Bari, Gavin Barlow, Stella Barnass, Nicholas Barrett, Christopher Bassford, Sneha Basude, David Baxter, Michael Beadsworth, Jolanta Bernatoniene, John Berridge, Nicola Best, Pieter Bothma, David Chadwick, Robin Brittain-Long, Naomi Bulteel, Tom Burden, Andrew Burtenshaw, Vikki Caruth, David Chadwick, Duncan Chambler, Nigel Chee, Jenny Child, Srikanth Chukkambotla, Tom Clark, Paul Collini, Catherine Cosgrove, Jason Cupitt, Maria-Teresa Cutino-Moguel, Paul Dark, Chris Dawson, Samir Dervisevic, Phil Donnison, Sam Douthwaite, Ingrid DuRand, Ahilanadan Dushianthan, Tristan Dyer, Cariad Evans, Chi Eziefula, Chrisopher Fegan, Adam Finn, Duncan Fullerton, Sanjeev Garg, Sanjeev Garg, Atul Garg, Effrossyni Gkrania-Klotsas, Jo Godden, Arthur Goldsmith, Clive Graham, Elaine Hardy, Stuart Hartshorn, Daniel Harvey, Peter Havalda, Daniel B Hawcutt, Maria Hobrok, Luke Hodgson, Anil Hormis, Michael Jacobs, Susan Jain, Paul Jennings, Agilan Kaliappan, Vidya Kasipandian, Stephen Kegg, Michael Kelsey, Jason Kendall, Caroline Kerrison, Ian Kerslake, Oliver Koch, Gouri Koduri, George Koshy, Shondipon Laha, Steven Laird, Susan Larkin, Tamas Leiner, Patrick Lillie, James Limb, Vanessa Linnett, Jeff Little, Mark Lyttle, Michael MacMahon, Emily MacNaughton, Ravish Mankregod, Huw Masson, Elijah Matovu, Katherine McCullough, Ruth McEwen, Manjula Meda, Gary Mills, Jane Minton, Mariyam Mirfenderesky, Kavya Mohandas, Quen Mok, James Moon, Elinoor Moore, Patrick Morgan, Craig Morris, Katherine Mortimore, Samuel Moses, Mbiye Mpenge, Rohinton Mulla, Michael Murphy,

For peer review only

Megan Nagel, Thapas Nagarajan, Mark Nelson, Matthew K. O'Shea, Igor Otahal, Marlies Ostermann, Mark Pais, Carlo Palmieri, Selva Panchatsharam, Danai Papakonstantinou, Hassan Paraiso, Brij Patel, Natalie Pattison, Justin Pepperell, Mark Peters, Mandeep Phull, Stefania Pintus, Jagtur Singh Pooni, Frank Post, David Price, Rachel Prout, Nikolas Rae, Henrik Reschreiter, Tim Reynolds, Neil Richardson, Mark Roberts, Devender Roberts, Alistair Rose, Guy Rousseau, Brendan Ryan, Taranprit Saluja, Aarti Shah, Prad Shanmuga, Anil Sharma, Anna Shawcross, Jeremy Sizer, Manu Shankar-Hari, Richard Smith, Catherine Snelson, Nick Spittle, Nikki Staines, Tom Stambach, Richard Stewart, Pradeep Subudhi, Tamas Szakmany, Kate Tatham, Jo Thomas, Chris Thompson, Robert Thompson, Ascanio Tridente, Darell Tupper-Carey, Mary Twagira, Andrew Ustianowski, Nick Vallotton, Lisa Vincent-Smith, Shico Visuvanathan, Alan Vuylsteke, Sam Waddy, Rachel Wake, Andrew Walden, Ingeborg Welters, Tony Whitehouse, Paul Whittaker, Ashley Whittington, Padmasayee Papineni, Meme Wijesinghe, Martin Williams, Lawrence Wilson, Sarah Cole, Stephen Winchester, Martin Wiselka, Adam Wolverson, Daniel G Wootton, Andrew Workman, Bryan Yates, Peter Young.

For peer review only

For peer review only

FIGURE CAPTIONS

Figure 1: Total 25(OH)D in COVID-19, influenza A and survivors of critical illness

(A) Total 25(OH)D concentrations in patients with COVID-19 (n=295) stratified by receipt of invasive mechanical ventilation. **(B)** Total 25(OH)D concentrations from COVID-19 patients were divided into quartiles and the proportion of patients who received IMV in each quartile was compared by Chi-squared test. **(C)** Total 25(OH)D concentrations in patients with influenza A (from 2009 H1N1 pandemic, n=93) stratified by receipt of invasive mechanical ventilation. For (A) and (C), groups are compared by Mann-Whitney test. The stacked bar charts represent the proportion of patients in each sub-group with sufficient (green), insufficient (orange), or deficient (red) total vitamin D status, compared by Chi-squared test. **(D)** Total 25(OH)D concentrations in non-selected critical illness survivors (n=139, recruited prior to the COVID-19 pandemic) at the time of ICU discharge. On violin plots of total 25(OH)D concentrations (nmol/L) the solid line within the plot represents the median and the dashed lines represent the interquartile range. The dotted lines on the y-axis represent the thresholds for total vitamin D insufficiency (25-50nmol/L) and deficiency (<25nmol/L).

Figure 2: Total and free 25(OH)D and outcomes in COVID-19 and influenza A

Smoothed predicted probability of outcomes (invasive mechanical ventilation or in-hospital mortality) vs. total or free 25(OH)D concentration (with other co-variates at mean values) from the binary logistic regression multivariable models. Grey ribbon represents estimated 95% confidence interval and the x-axis ticks show observations.

Figure 3: Free 25(OH)D in COVID-19

(A) Simple linear regression line and 95% confidence interval (dashed lines) representing the correlation between total and free 25(OH)D concentrations in COVID-19. (B) Violin plot of free 25(OH)D concentrations (pg/ml) in patients with COVID-19 stratified by receipt of invasive mechanical

For peer review only - http://bmjopen.bmj.com/site/about/guidelines.xhtml

ventilation. The solid line within the plot represents the median and the dashed lines represent the interquartile range. Groups are compared by Mann-Whitney test.

For peer review only

For peer review only - http://bmjopen.bmj.com/site/about/guidelines.xhtml

REFERENCES

1. Hewison M. Vitamin D and the intracrinology of innate immunity. Mol Cell Endocrinol. 2010;321(2):103-11.
2. Chun RF, Lauridsen AL, Suon L, Zella LA, Pike JW, Modlin RL, et al. Vitamin D-binding protein directs monocyte responses to 25-hydroxy- and 1,25-dihydroxyvitamin D. J Clin Endocrinol Metab. 2010;95(7):3368-76.
3. Sabetta JR, DePetrillo P, Cipriani RJ, Smardin J, Burns LA, Landry ML. Serum 25-hydroxyvitamin d and the incidence of acute viral respiratory tract infections in healthy adults. PLoS One. 2010;5(6):e11088.
4. Monlezun DJ, Bittner EA, Christopher KB, Camargo CA, Quraishi SA. Vitamin D status and acute respiratory infection: cross sectional results from the United States National Health and Nutrition Examination Survey, 2001-2006. Nutrients. 2015;7(3):1933-44.
5. Dancer RC, Parekh D, Lax S, D'Souza V, Zheng S, Bassford CR, et al. Vitamin D deficiency contributes directly to the acute respiratory distress syndrome (ARDS). Thorax. 2015;70(7):617-24.
6. Meltzer DO, Best TJ, Zhang H, Vokes T, Arora V, Solway J. Association of Vitamin D Status and Other Clinical Characteristics With COVID-19 Test Results. JAMA Netw Open. 2020;3(9):e2019722.
7. Kaufman HW, Niles JK, Kroll MH, Bi C, Holick MF. SARS-CoV-2 positivity rates associated with circulating 25-hydroxyvitamin D levels. PLoS One. 2020;15(9):e0239252.
8. Papadimitriou DT, Vassaras AK, Holick MF. Association between population vitamin D status and SARS-CoV-2 related serious-critical illness and deaths: An ecological integrative approach. World J Virol. 2021;10(3):111-29.
9. Quraishi SA, Camargo CA, Jr. Vitamin D in acute stress and critical illness. Curr Opin Clin Nutr Metab Care. 2012;15(6):625-34.
10. Mathyssen C, Gayan-Ramirez G, Bouillon R, Janssens W. Vitamin D supplementation in respiratory diseases: evidence from randomized controlled trials. Pol Arch Intern Med. 2017;127(11):775-84.
11. Martineau AR, Jolliffe DA, Greenberg L, Aloia JF, Bergman P, Dubnov-Raz G, et al. Vitamin D supplementation to prevent acute respiratory infections: individual participant data meta-analysis. Health Technol Assess. 2019;23(2):1-44.
12. Ganmaa D, Uyanga B, Zhou X, Gantsetseg G, Delgerekh B, Enkhmaa D, et al. Vitamin D Supplements for Prevention of Tuberculosis Infection and Disease. N Engl J Med. 2020;383(4):359-68.
13. Murai IH, Fernandes AL, Sales LP, Pinto AJ, Goessler KF, Duran CSC, et al. Effect of a Single High Dose of Vitamin D3 on Hospital Length of Stay in Patients With Moderate to Severe COVID-19: A Randomized Clinical Trial. Jama. 2021;325(11):1053-60.
14. Orford NR, Lane SE, Bailey M, Pasco JA, Cattigan C, Elderkin T, et al. Changes in Bone Mineral Density in the Year after Critical Illness. Am J Respir Crit Care Med. 2016;193(7):736-44.
15. Docherty AB, Harrison EM, Green CA, Hardwick HE, Pius R, Norman L, et al. Features of 20 133 UK patients in hospital with covid-19 using the ISARIC WHO Clinical Characterisation Protocol: prospective observational cohort study. Bmj. 2020;369:m1985.
16. Walsh TS, Salisbury LG, Merriweather JL, Boyd JA, Griffith DM, Huby G, et al. Increased Hospital-Based Physical Rehabilitation and Information Provision After Intensive Care Unit Discharge: The RECOVER Randomized Clinical Trial. JAMA Intern Med. 2015;175(6):901-10.

For peer review only - http://bmjopen.bmj.com/site/about/guidelines.xhtml

17. Griffith DM, Lewis S, Rossi AG, Rennie J, Salisbury L, Merriweather JL, et al. Systemic inflammation after critical illness: relationship with physical recovery and exploration of potential mechanisms. Thorax. 2016;71(9):820-9.
18. Dunning J, Blankley S, Hoang LT, Cox M, Graham CM, James PL, et al. Progression of whole-blood transcriptional signatures from interferon-induced to neutrophil-associated patterns in severe influenza. Nat Immunol. 2018;19(6):625-35.
19. Hurst EA, Homer NZ, Gow AG, Clements DN, Evans H, Gaylor D, et al. Vitamin D status is seasonally stable in northern European dogs. Vet Clin Pathol. 2020;49(2):279-91.
20. Sai AJ, Walters RW, Fang X, Gallagher JC. Relationship between vitamin D, parathyroid hormone, and bone health. J Clin Endocrinol Metab. 2011;96(3):E436-46.
21. Wood SN. Thin plate regression splines. Journal of the Royal Statistical Society: Series B (Statistical Methodology). 2003;65(1):95-114.
22. Wood SN. Fast stable restricted maximum likelihood and marginal likelihood estimation of semiparametric generalized linear models. Journal of the Royal Statistical Society: Series B (Statistical Methodology). 2011;73(1):3-36.
23. Lubin JH, Colt JS, Camann D, Davis S, Cerhan JR, Severson RK, et al. Epidemiologic evaluation of measurement data in the presence of detection limits. Environ Health Perspect. 2004;112(17):1691-6.
24. Thwaites RS, Sanchez Sevilla Uruchurtu A, Siggins MK, Liew F, Russell CD, Moore SC, et al. Inflammatory profiles across the spectrum of disease reveal a distinct role for GM-CSF in severe COVID-19. Sci Immunol. 2021;6(57).
25. D'Avolio A, Avataneo V, Manca A, Cusato J, De Nicolò A, Lucchini R, et al. 25-Hydroxyvitamin D Concentrations Are Lower in Patients with Positive PCR for SARS-CoV-2. Nutrients. 2020;12(5).
26. Panagiotou G, Tee SA, Ihsan Y, Athar W, Marchitelli G, Kelly D, et al. Low serum 25-hydroxyvitamin D (25[OH]D) levels in patients hospitalized with COVID-19 are associated with greater disease severity. Clin Endocrinol (Oxf). 2020;93(4):508-11.
27. Carpagnano GE, Di Lecce V, Quaranta VN, Zito A, Buonamico E, Capozza E, et al. Vitamin D deficiency as a predictor of poor prognosis in patients with acute respiratory failure due to COVID-19. J Endocrinol Invest. 2021;44(4):765-71.
28. Yuk JM, Shin DM, Lee HM, Yang CS, Jin HS, Kim KK, et al. Vitamin D3 induces autophagy in human monocytes/macrophages via cathelicidin. Cell Host Microbe. 2009;6(3):231-43.
29. Liu PT, Stenger S, Li H, Wenzel L, Tan BH, Krutzik SR, et al. Toll-like receptor triggering of a vitamin D-mediated human antimicrobial response. Science. 2006;311(5768):1770-3.
30. Campbell GR, Spector SA. Hormonally active vitamin D3 (1alpha,25-dihydroxycholecalciferol) triggers autophagy in human macrophages that inhibits HIV-1 infection. J Biol Chem. 2011;286(21):18890-902.
31. Khare D, Godbole NM, Pawar SD, Mohan V, Pandey G, Gupta S, et al. Calcitriol [1, 25[OH]2 D3] pre- and post-treatment suppresses inflammatory response to influenza A (H1N1) infection in human lung A549 epithelial cells. Eur J Nutr. 2013;52(4):1405-15.
32. Chun RF, Peercy BE, Adams JS, Hewison M. Vitamin D binding protein and monocyte response to 25-hydroxyvitamin D and 1,25-dihydroxyvitamin D: analysis by mathematical modeling. PLoS One. 2012;7(1):e30773.
33. Larner DP, Jenkinson C, Chun RF, Westgate CSJ, Adams JS, Hewison M. Free versus total serum 25-hydroxyvitamin D in a murine model of colitis. J Steroid Biochem Mol Biol. 2019;189:204-9.

For peer review only - http://bmjopen.bmj.com/site/about/guidelines.xhtml

34. Han JE, Alvarez JA, Jones JL, Tangpricha V, Brown MA, Hao L, et al. Impact of high-dose vitamin D(3) on plasma free 25-hydroxyvitamin D concentrations and antimicrobial peptides in critically ill mechanically ventilated adults. Nutrition. 2017;38:102-8.
35. Quraishi SA, De Pascale G, Needleman JS, Nakazawa H, Kaneki M, Bajwa EK, et al. Effect of Cholecalciferol Supplementation on Vitamin D Status and Cathelicidin Levels in Sepsis: A Randomized, Placebo-Controlled Trial. Crit Care Med. 2015;43(9):1928-37.
36. Braun A, Chang D, Mahadevappa K, Gibbons FK, Liu Y, Giovannucci E, et al. Association of low serum 25-hydroxyvitamin D levels and mortality in the critically ill. Crit Care Med. 2011;39(4):671-7.
37. Lucidarme O, Messai E, Mazzoni T, Arcade M, du Cheyron D. Incidence and risk factors of vitamin D deficiency in critically ill patients: results from a prospective observational study. Intensive Care Med. 2010;36(9):1609-11.
38. Czarnik T, Czarnik A, Gawda R, Gawor M, Piwoda M, Marszalski M, et al. Vitamin D kinetics in the acute phase of critical illness: A prospective observational study. J Crit Care. 2018;43:294-9.
39. Amrein K, Christopher KB, McNally JD. Understanding vitamin D deficiency in intensive care patients. Intensive Care Med. 2015;41(11):1961-4.
40. Lee P. Vitamin D metabolism and deficiency in critical illness. Best Pract Res Clin Endocrinol Metab. 2011;25(5):769-81.
41. Braun AB, Litonjua AA, Moromizato T, Gibbons FK, Giovannucci E, Christopher KB. Association of low serum 25-hydroxyvitamin D levels and acute kidney injury in the critically ill. Crit Care Med. 2012;40(12):3170-9.
42. Arnson Y, Gringauz I, Itzhaky D, Amital H. Vitamin D deficiency is associated with poor outcomes and increased mortality in severely ill patients. Qjm. 2012;105(7):633-9.
43. de Haan K, Groeneveld AB, de Geus HR, Egal M, Struijs A. Vitamin D deficiency as a risk factor for infection, sepsis and mortality in the critically ill: systematic review and meta-analysis. Crit Care. 2014;18(6):660.
44. Malabanan A, Veronikis IE, Holick MF. Redefining vitamin D insufficiency. Lancet. 1998;351(9105):805-6.
45. Amrein K, Schnedl C, Holl A, Riedl R, Christopher KB, Pachler C, et al. Effect of high-dose vitamin D3 on hospital length of stay in critically ill patients with vitamin D deficiency: the VITdAL-ICU randomized clinical trial. Jama. 2014;312(15):1520-30.
46. Ginde AA, Brower RG, Caterino JM, Finck L, Banner-Goodspeed VM, Grissom CK, et al. Early High-Dose Vitamin D(3) for Critically Ill, Vitamin D-Deficient Patients. N Engl J Med. 2019;381(26):2529-40.
47. Entrenas Castillo M, Entrenas Costa LM, Vaquero Barrios JM, Alcalá Díaz JF, López Miranda J, Bouillon R, et al. "Effect of calcifediol treatment and best available therapy versus best available therapy on intensive care unit admission and mortality among patients hospitalized for COVID-19: A pilot randomized clinical study". J Steroid Biochem Mol Biol. 2020;203:105751.
48. Nogues X, Ovejero D, Pineda-Moncusí M, Bouillon R, Arenas D, Pascual J, et al. Calcifediol treatment and COVID-19-related outcomes. J Clin Endocrinol Metab. 2021.
49. Ma H, Zhou T, Heianza Y, Qi L. Habitual use of vitamin D supplements and risk of coronavirus disease 2019 (COVID-19) infection: a prospective study in UK Biobank. Am J Clin Nutr. 2021;113(5):1275-81.
50. Ney J, Heyland DK, Amrein K, Marx G, Grottke O, Choudrakis M, et al. The relevance of 25-hydroxyvitamin D and 1,25-dihydroxyvitamin D concentration for postoperative

For peer review only

infections and postoperative organ dysfunctions in cardiac surgery patients: The eVIDenCe study. Clin Nutr. 2019;38(6):2756-62.

For peer review only

For peer review only - http://bmjopen.bmj.com/site/about/guidelines.xhtml

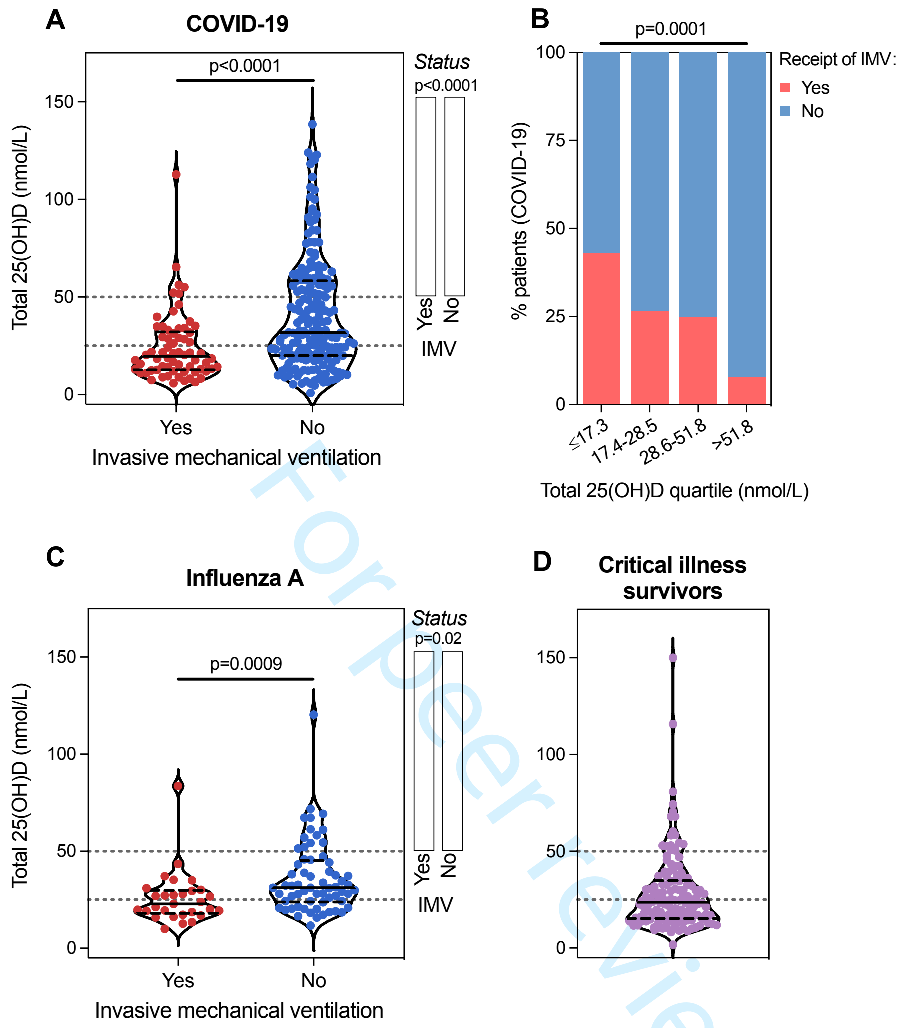

Figure 1: Total 25(OH)D in COVID-19, influenza A and survivors of critical illness

(A) Total 25(OH)D concentrations in patients with COVID-19 (n=295) stratified by receipt of invasive mechanical ventilation. **(B)** Total 25(OH)D concentrations from COVID-19 patients were divided into quartiles and the proportion of patients who received IMV in each quartile was compared by Chi-squared test. **(C)** Total 25(OH)D concentrations in patients with influenza A (from 2009 H1N1 pandemic, n=93) stratified by receipt of invasive mechanical ventilation. For (A) and (C), groups are compared by Mann-Whitney test. The stacked bar charts represent the proportion of patients in each sub-group with sufficient (green), insufficient (orange), or deficient (red) total vitamin D status, compared by Chi-squared test. **(D)** Total 25(OH)D concentrations in non-selected critical illness survivors (n=139, recruited prior to the COVID-19 pandemic) at the time of ICU discharge. On violin

plots of total 25(OH)D concentrations (nmol/L) the solid line within the plot represents the median and the dashed lines represent the interquartile range. The dotted lines on the y-axis represent the thresholds for total vitamin D insufficiency (25-50nmol/L) and deficiency (<25nmol/L).

For peer review only

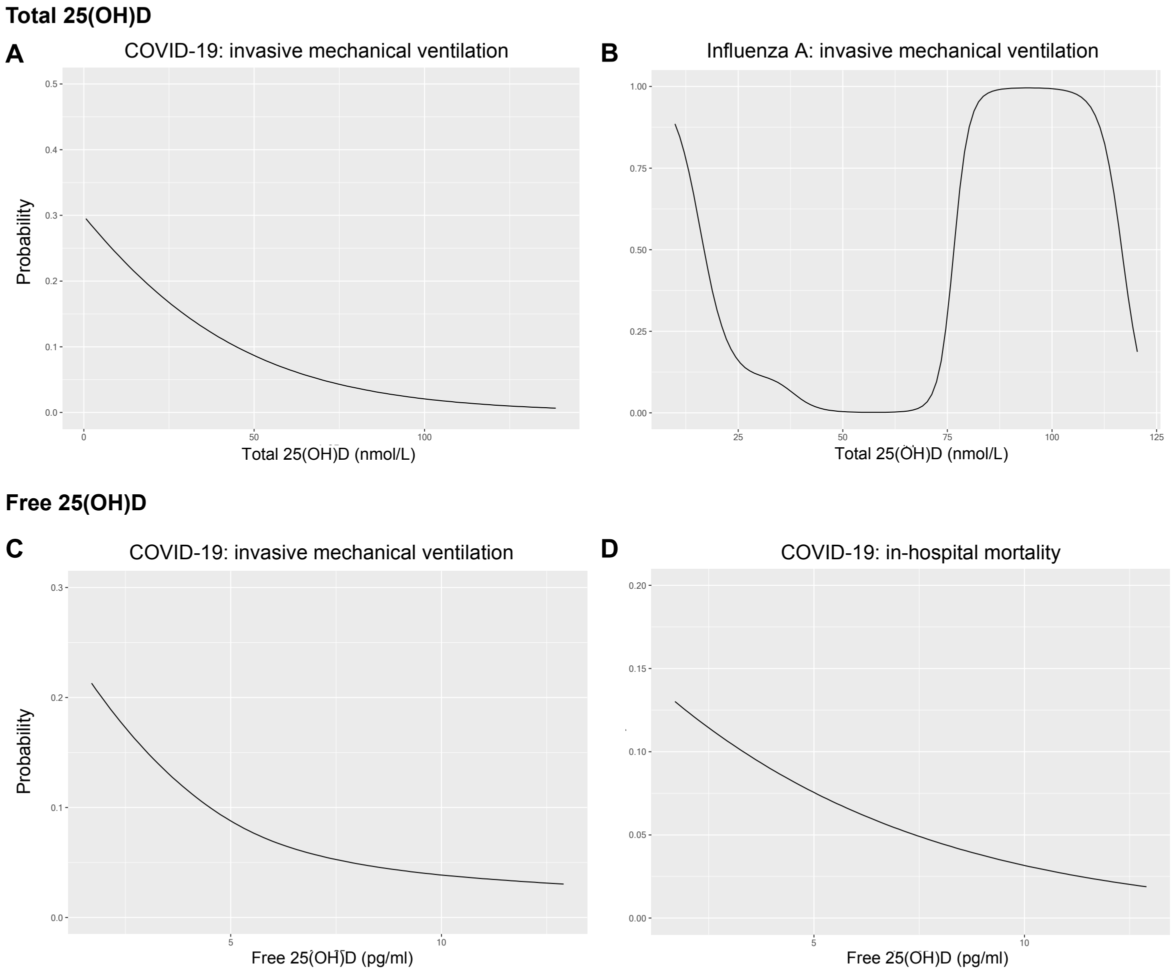

Figure 2: Total and free 25(OH)D and outcomes in COVID-19 and influenza A

Smoothed predicted probability of outcomes (invasive mechanical ventilation or in-hospital mortality) vs. total or free 25(OH)D concentration (with other co-variates at mean values) from the binary logistic regression multivariable models. Grey ribbon represents estimated 95% confidence interval and the x-axis ticks show observations.

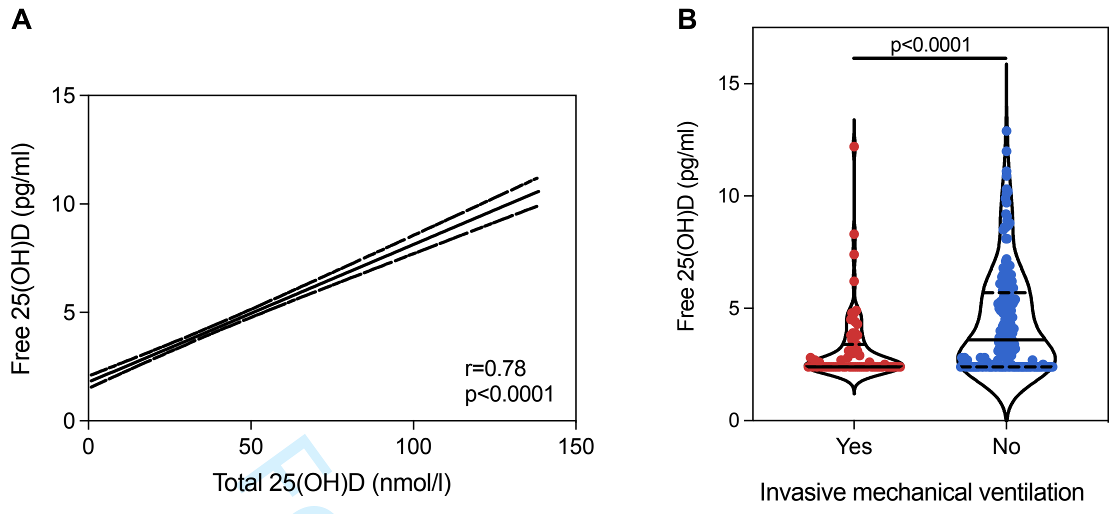

Figure 3: Free 25(OH)D in COVID-19

(A) Simple linear regression line and 95% confidence interval (dashed lines) representing the correlation between total and free 25(OH)D concentrations in COVID-19. **(B)** Violin plot of free 25(OH)D concentrations (pg/ml) in patients with COVID-19 stratified by receipt of invasive mechanical ventilation. The solid line within the plot represents the median and the dashed lines represent the interquartile range. Groups are compared by Mann-Whitney test.

For peer review only

SUPPLEMENTARY DATA

Supplementary Methods

Definition of vitamin D status

A total 25(OH)D concentration of 50nmol/L is the value widely used to define vitamin D sufficiency since experimental studies have shown that this is the concentration at which parathyroid hormone concentrations plateau [1,2]. Furthermore, based on evidence from the Institute of Medicine (IOM) and the Scientific Advisory Committee on Nutrition (SACN) which demonstrate an increased risk of poor muscoskeletal health with 25(OH)D levels between 20-30 nmol/L, the Royal Osteoporosis Society guidelines (which advice on testing and treatment of vitamin D in primary care in for the NHS), suggest that plasma 25(OH)D of 25-50 nmol/L may be inadequate in some people [3].

1. Sai AJ, Walters RW, Fang X, Gallagher JC. Relationship between Vitamin D, Parathyroid Hormone, and Bone Health. J Clin Endocrinol Metab **2011**; 96:E436–E446. Available at: https://doi.org/10.1210/jc.2010-1886.
2. Lips P. Vitamin D Deficiency and Secondary Hyperparathyroidism in the Elderly: Consequences for Bone Loss and Fractures and Therapeutic Implications. Endocr Rev **2001**; 22:477–501. Available at: https://doi.org/10.1210/edrv.22.4.0437.
3. Francis R, Aspray T, Fraser W, et al. Vitamin D and Bone Health : A Practical Clinical Guideline for Patient Management. 2018. Available at: https://strwebprdmedia.blob.core.windows.net/media/ef2ideu2/ros-vitamin-d-and-bone-health-in-adults-february-2020.pdf.

For peer review only

Supplementary Table 1: LC-MS/MS method parameters

**Parameter**	**COVID-19** (n=259) **/ ICU** (n=139) **sample method**	**Influenza A** (n=93) **/ healthy controls** (n=36) **sample method**
**Sample preparation**		
Isotopically labelled internal standards	d_3-25(OH)D2<br>$^{13}C_5$-25(OH)D3	-<br>d_6-25(OH)D3
Extraction method	Automated SLE	PPT + LLE
Derivatization	DMEQ-TAD	-
**LC-MS instrumentation**		
LC-MS system	Shimadzu Nexera UPLC – Sciex QTrap 6500+	Waters ACQUITY TQD UPLC/MS/MS
LC column	Raptor Fluorophenyl column (2.7µm 100 Å, 100 x 2.1 mm)	Phenyl reversed phase LC column
Ionization mode	ESI, positive	Turbulon Spray, positive
Detection mode	MRM	MRM
**Method specifications**		
LLOD	0.5 nmol/L 25(OH)D2<br>4 nmol/L 25(OH)D3	10 nmol/L 25(OH)D2<br>10 nmol/L 25(OH)D3
Inter-assay precision (CV)	<11.5% 25(OH)D2<br><11.5% 25(OH)D3	<11% 25(OH)D2<br><10% 25(OH)D3

n: number of patient samples analysed; d: deuterium labelled; ^{13}C: carbon 13 labelled; SLE: supported liquid extraction performed on the Biotage® Extrahera™; PPT: protein precipitation; LLE: liquid liquid extraction using n-hexane; ESI: electrospray ionization; MRM: multiple reaction monitoring; LLOD: lower limit of detection; CV: coefficient of variation.

For peer review only

Supplementary Table 2: Multivariable analyses of total 25(OH)D and vitamin D status and outcomes in COVID-19

Variable	Odds ratio	p-value
**Vitamin D status**		
Receipt of IMV		
**Sufficient**[a]	0.26 (0.1-0.62)	**0.004**
Male sex	2.41 (1.18-4.91)	**0.015**
Comorbidity count[b]	-	0.805
Day of illness[b]	-	0.462
Age[b]	-	**0.018**
In-hospital mortality		
**Sufficient**[a]	0.27 (0.11-0.68)	**0.005**
Male sex	2.52 (1.13-5.63)	**0.024**
Comorbidity count[b]		**0.016**
Day of illness[b]	-	0.576
Age[b]	-	0.059
**Total 25(OH)D concentration**		
In-hospital mortality		
Male sex	2.49 (1.12-5.57)	**0.026**
Comorbidity count[b]	-	**0.030**
**Total 25(OH)D**[b]	-	0.068
Day of illness[b]	-	0.495
Age[b]	-	**0.046**

[a]total 25(OH)D >50 nmol/l
[b]smoothed

Supplementary Table 3: Multivariable analysis of total 25(OH)D concentration and in-hospital mortality in influenza A

Variable	Odds ratio	p-value
In-hospital mortality		
Male sex	0.84	0.798
Comorbidity count[a]	-	0.539
**Total 25(OH)D**[a]	-	0.421
Day of illness[a]	-	0.244
Age[a]	-	0.200

[a]smoothed

For peer review only

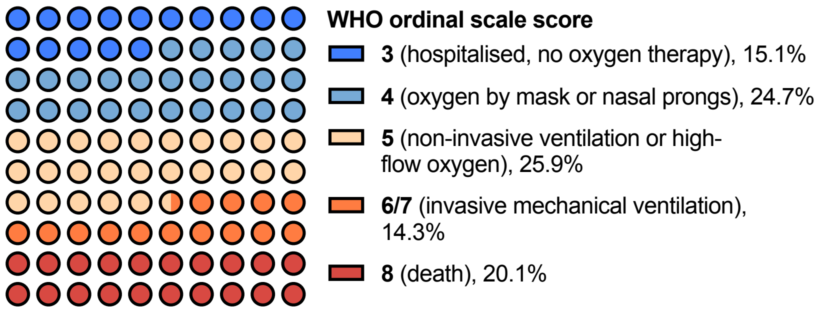

Supplementary Figure 1: WHO COVID-19 ordinal severity scale scores

The % refers to the % of patients in the cohort (n=259) with the score. Scores represent maximum illness severity during the hospital admission.

For peer review only

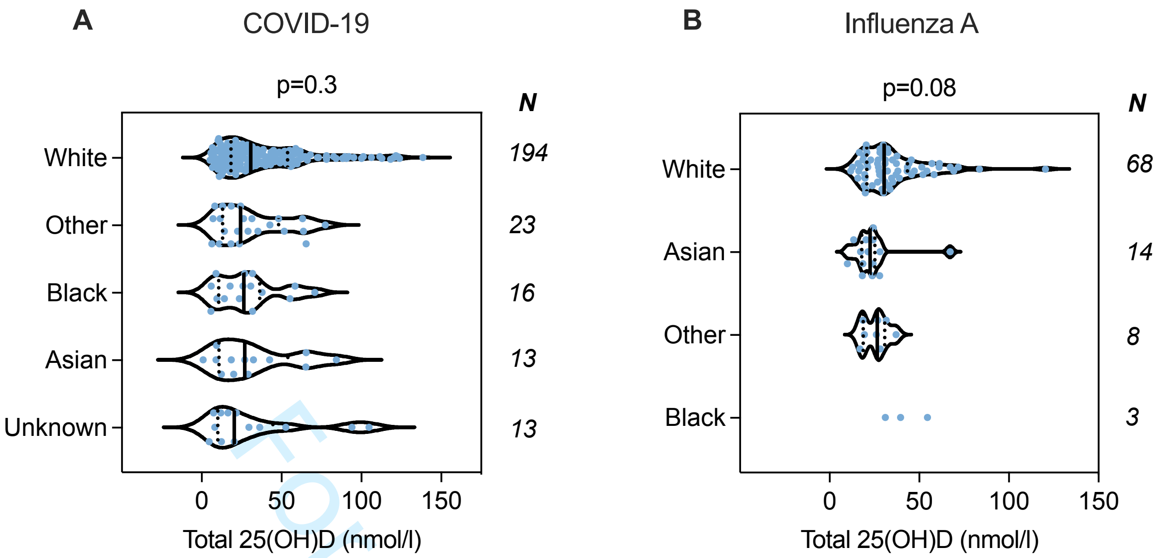

Supplementary Figure 2: Total 25(OH)D concentration stratified by ethnicity
(A) COVID-19 and **(B)** influenza A. The solid line within the violin plot represents the median and the dotted lines represent the interquartile range. Groups ≤5 are shown as individual data points. Groups were compared by ANOVA. N refers to the number of patients in each group.

For peer review only

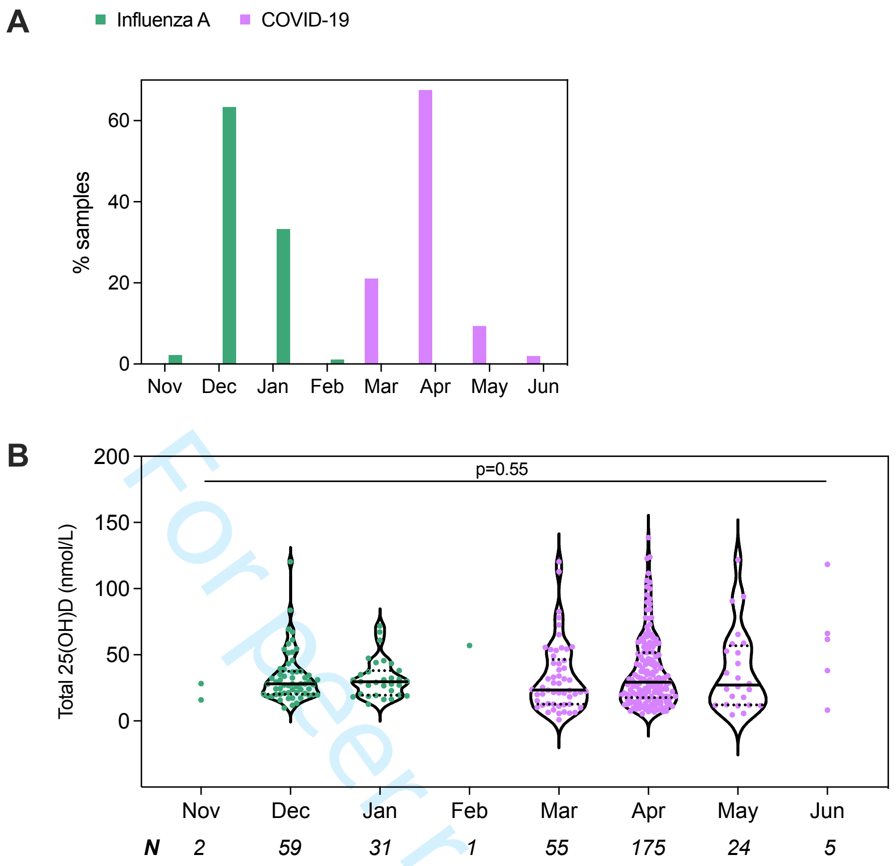

Supplementary Figure 3: Total 25(OH)D stratified by months of the year
(A) Month of the year during which samples were obtained from people with influenza A (2009-2011) and COVID-19 (2020). **(B)** Total 25(OH)D concentrations stratified by month of the year the sample was obtained. Groups compared by Kruskal-Wallis test. The solid line within the violin plot represents the median and the dotted lines represent the interquartile range. N refers to the number of samples for each month. Groups ≤5 are shown as individual data points.

For peer review only

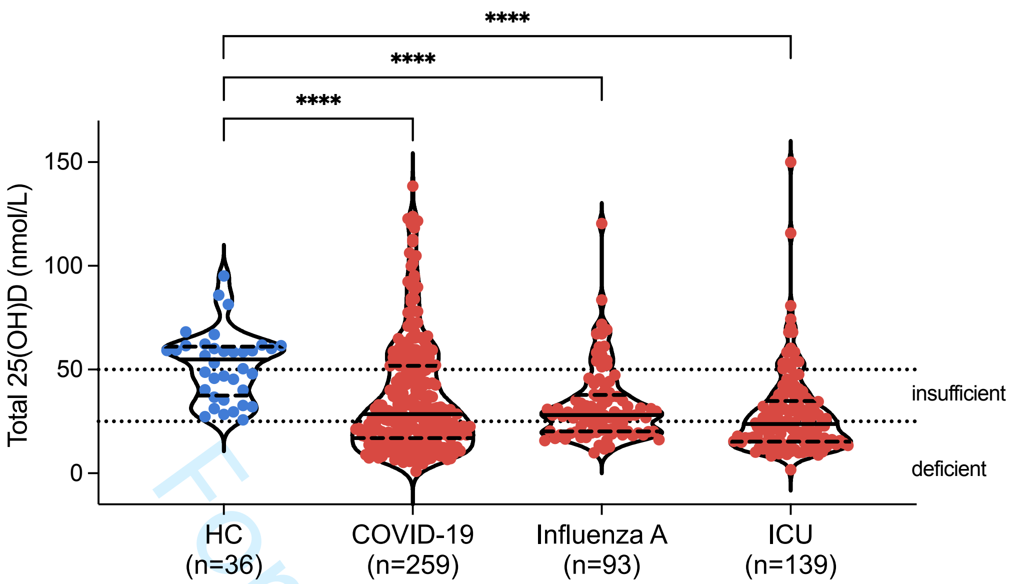

Supplementary Figure 4: Total 25(OH)D in patient cohorts compared to healthy controls
Total 25(OH)D measured in healthy controls ("HC", from the MOSAIC study recruited between June-September 2011), hospitalised patients with COVID-19 and influenza A, and non-selected critical illness survivors ("ICU"). The solid line within the violin plot represents the median and the dashed lines represent the interquartile range. Patient cohorts compared to healthy controls by Kruskal-Wallis test and Dunn's multiple comparisons test. **** p<0.0001

For peer review only

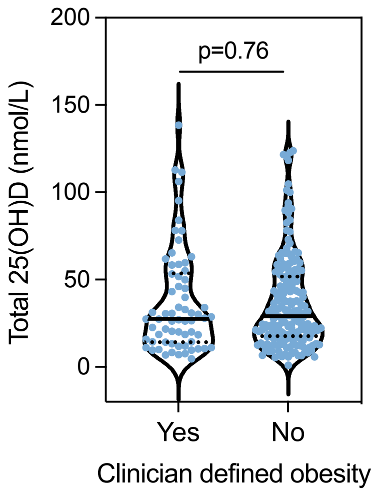

Supplementary Figure 5: Total 25(OH)D in patients with COVID-19 with/without obesity
Total 25(OH)D levels in hospitalised patients with COVID-19 with/without clinician defined obesity. Groups compared by Mann-Whitney test. The solid line within the violin plot represents the median and the dotted lines represent the interquartile range.

For peer review only

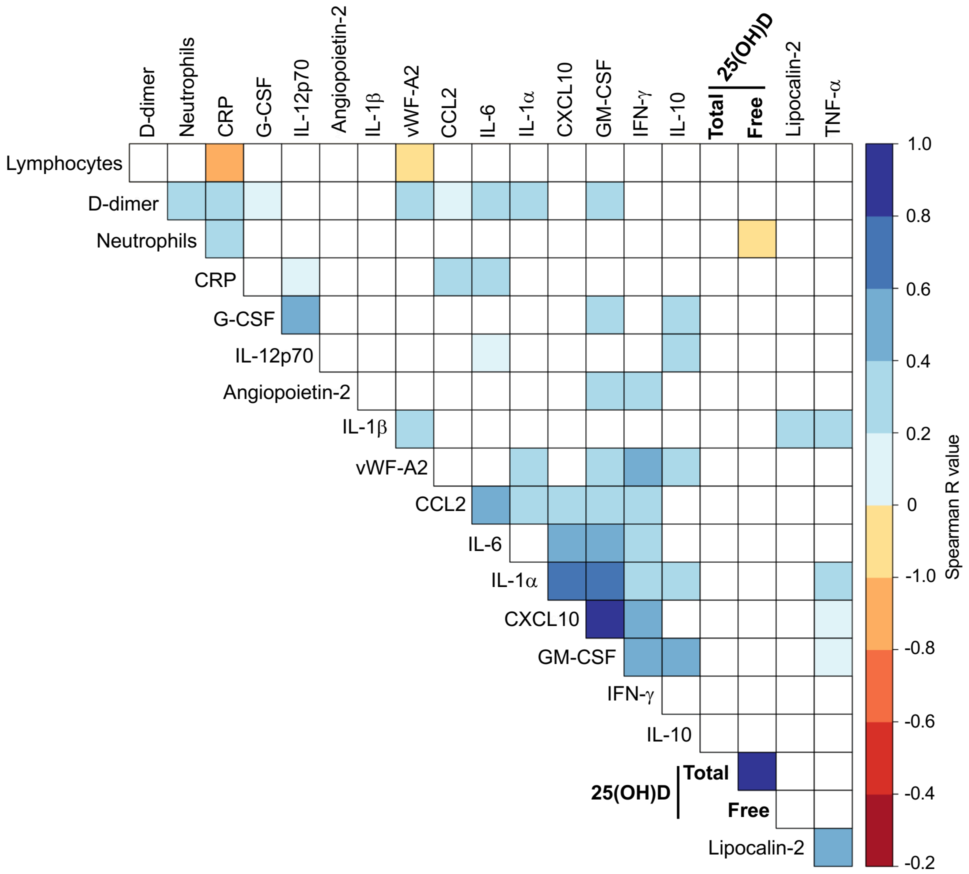

Supplementary Figure 6: Correlation analysis of 25(OH)D and inflammatory mediators
Correlogram of concentrations of plasma inflammatory mediators associated with COVID-19 severity and 25(OH)D (free and total). Cells with a correlation with $p<0.05$ (after correction for multiple comparisons) are shaded according to the Spearman R value. Inflammatory mediator measurements were available for 66 patients. Analysis was performed using the *corrplot* package in R.
CRP: C-reactive protein; G-CSF: granulocyte colony-stimulating factor; IL: interleukin; vWF: von Willebrand Factor; CCL2: C-C Motif Chemokine Ligand 2; CXCL10: C-X-C Motif Chemokine Ligand 10; GM-CSF: granulocyte-macrophage colony-stimulating factor; IFN-γ: interferon gamma; TNF-α: tumour necrosis factor alpha.

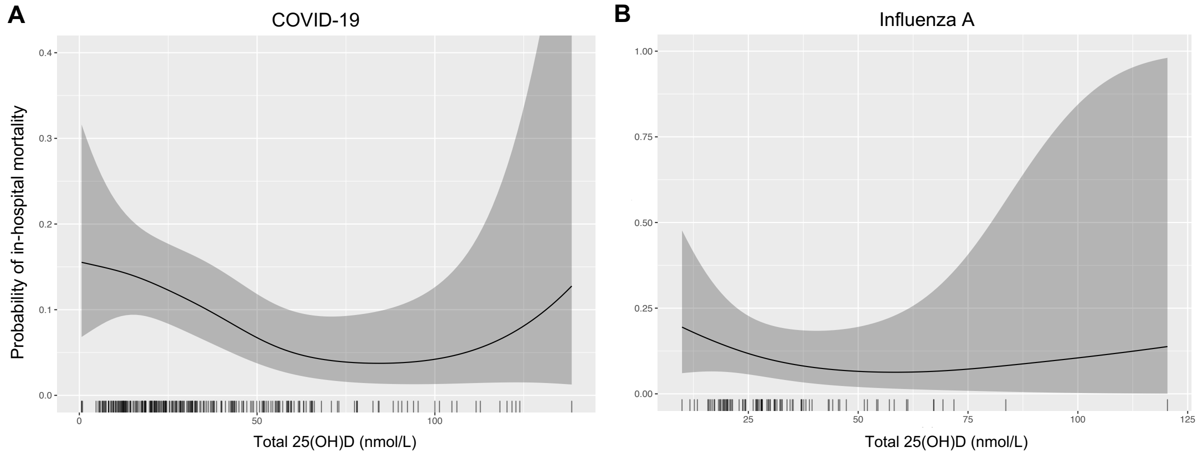

Supplementary Figure 7: Total 25(OH)D concentration and in-hospital mortality in COVID-19 and influenza A.

Smoothed predicted probability of in-hospital mortality vs. total 25(OH)D concentration (with other co-variates at mean values) from the binary logistic regression multivariable models for hospitalised people with **(A)** COVID-19 and **(B)** influenza A. Grey ribbon represents estimated 95% confidence interval and the x-axis ticks show observations.

STROBE 2007 (v4) Statement—Checklist of items that should be included in reports of *cross-sectional studies*

Section/Topic	Item #	Recommendation	Reported on page #
**Title and abstract**	1	(*a*) Indicate the study's design with a commonly used term in the title or the abstract	1
		(*b*) Provide in the abstract an informative and balanced summary of what was done and what was found	3
**Introduction**			
Background/rationale	2	Explain the scientific background and rationale for the investigation being reported	5
Objectives	3	State specific objectives, including any prespecified hypotheses	5-6
**Methods**			
Study design	4	Present key elements of study design early in the paper	5
Setting	5	Describe the setting, locations, and relevant dates, including periods of recruitment, exposure, follow-up, and data collection	7-8
Participants	6	(*a*) Give the eligibility criteria, and the sources and methods of selection of participants	7-8
Variables	7	Clearly define all outcomes, exposures, predictors, potential confounders, and effect modifiers. Give diagnostic criteria, if applicable	9
Data sources/ measurement	8*	For each variable of interest, give sources of data and details of methods of assessment (measurement). Describe comparability of assessment methods if there is more than one group	8-9
Bias	9	Describe any efforts to address potential sources of bias	NA
Study size	10	Explain how the study size was arrived at	NA
Quantitative variables	11	Explain how quantitative variables were handled in the analyses. If applicable, describe which groupings were chosen and why	9
Statistical methods	12	(*a*) Describe all statistical methods, including those used to control for confounding	9
		(*b*) Describe any methods used to examine subgroups and interactions	NA
		(*c*) Explain how missing data were addressed	No missing data
		(*d*) If applicable, describe analytical methods taking account of sampling strategy	9-10
		(*e*) Describe any sensitivity analyses	NA
**Results**			

For peer review only

Participants	13*	(a) Report numbers of individuals at each stage of study—eg numbers potentially eligible, examined for eligibility, confirmed eligible, included in the study, completing follow-up, and analysed	11
		(b) Give reasons for non-participation at each stage	NA
		(c) Consider use of a flow diagram	NA
Descriptive data	14*	(a) Give characteristics of study participants (eg demographic, clinical, social) and information on exposures and potential confounders	11, Table 1
		(b) Indicate number of participants with missing data for each variable of interest	None
Outcome data	15*	Report numbers of outcome events or summary measures	11, Table 1
Main results	16	(*a*) Give unadjusted estimates and, if applicable, confounder-adjusted estimates and their precision (eg, 95% confidence interval). Make clear which confounders were adjusted for and why they were included	Yes, throughout
		(*b*) Report category boundaries when continuous variables were categorized	8-9
		(*c*) If relevant, consider translating estimates of relative risk into absolute risk for a meaningful time period	NA
Other analyses	17	Report other analyses done—eg analyses of subgroups and interactions, and sensitivity analyses	NA
**Discussion**			
Key results	18	Summarise key results with reference to study objectives	18
Limitations	19	Discuss limitations of the study, taking into account sources of potential bias or imprecision. Discuss both direction and magnitude of any potential bias	20
Interpretation	20	Give a cautious overall interpretation of results considering objectives, limitations, multiplicity of analyses, results from similar studies, and other relevant evidence	20
Generalisability	21	Discuss the generalisability (external validity) of the study results	20
**Other information**			
Funding	22	Give the source of funding and the role of the funders for the present study and, if applicable, for the original study on which the present article is based	21-22

*Give information separately for cases and controls in case-control studies and, if applicable, for exposed and unexposed groups in cohort and cross-sectional studies.

Note: An Explanation and Elaboration article discusses each checklist item and gives methodological background and published examples of transparent reporting. The STROBE checklist is best used in conjunction with this article (freely available on the Web sites of PLoS Medicine at http://www.plosmedicine.org/, Annals of Internal Medicine at http://www.annals.org/, and Epidemiology at http://www.epidem.com/). Information on the STROBE Initiative is available at www.strobe-statement.org.

For peer review only
